# Retracing the evolution of a modern periplasmic binding protein

**DOI:** 10.1002/pro.4793

**Published:** 2023-10-26

**Authors:** Florian Michel, Sergio Romero‐Romero, Birte Höcker

**Affiliations:** ^1^ Department of Biochemistry University of Bayreuth Bayreuth Germany

**Keywords:** flavodoxin‐like fold, gene duplication, protein evolution, ribose binding protein, solute binding protein

## Abstract

Investigating the evolution of structural features in modern multidomain proteins helps to understand their immense diversity and functional versatility. The class of periplasmic binding proteins (PBPs) offers an opportunity to interrogate one of the main processes driving diversification: the duplication and fusion of protein sequences to generate new architectures. The symmetry of their two‐lobed topology, their mechanism of binding, and the organization of their operon structure led to the hypothesis that PBPs arose through a duplication and fusion event of a single common ancestor. To investigate this claim, we set out to reverse the evolutionary process and recreate the structural equivalent of a single‐lobed progenitor using ribose‐binding protein (RBP) as our model. We found that this modern PBP can be deconstructed into its lobes, producing two proteins that represent possible progenitor halves. The isolated halves of RBP are well folded and monomeric proteins, albeit with a lower thermostability, and do not retain the original binding function. However, the two entities readily form a heterodimer *in vitro* and *in‐cell*. The x‐ray structure of the heterodimer closely resembles the parental protein. Moreover, the binding function is fully regained upon formation of the heterodimer with a ligand affinity similar to that observed in the modern RBP. This highlights how a duplication event could have given rise to a stable and functional PBP‐like fold and provides insights into how more complex functional structures can evolve from simpler molecular components.

## 1 INTRODUCTION

The detection of chemicals in the environment, their molecular recognition and transport into the cell as well as the resulting downstream signaling is an integral part of life in any cell. As one of the central classes of proteins responsible for this function in prokaryotes, the periplasmic binding proteins (PBPs) serve as an important element in these complex response networks (Matilla et al., 2021). These bilobal proteins are involved in the transport of a wide variety of substrates, and are generally considered to belong to an ancient protein fold (Clifton & Jackson, 2016; Felder et al., 1999).

The PBP architecture consists of two opposing lobes, with each lobe being built of a central, five‐stranded parallel β‐sheet with five α‐helices flanking its sides. The two lobes are connected via a hinge region, with the complexity and number of crossovers dependent on the class of PBP. This architecture also gives rise to the most common mechanism in which PBPs recognize and bind their ligands (Berntsson et al., 2010; Chandravanshi et al., 2021; Scheepers et al., 2016). This distinct mode of binding that a majority of PBPs follow is a “venus flytrap‐like” mechanism and considered one of the hallmark features of this protein class (Felder et al., 1999). While in the unbound state, PBPs are in an “open” form with a space created by the two lobes accessible to surrounding solutes. Recognition and binding of the ligand facilitates interaction between the two lobes, leading to the eponymous hinge‐bending motion which results in the “closed” conformation with the cleft now being tightly shut around the ligand, excluding the solvent upon binding (Berntsson et al., 2010; Felder et al., 1999). This common binding mechanism is reflected in PBPs that bind similar molecules with very different selectivities and affinities at the same binding site (Kröger, Shanmugaratnam, Ferruz, et al., 2021). For these reasons, PBPs have been used in several engineering and design approaches, especially creating highly sensitive biosensors and molecular switches (Dwyer & Hellinga, 2004; Jeffery, 2011; Medintz & Deschamps, 2006; Steffen et al., 2016), and designing new binding properties (Banda‐Vázquez et al., 2018; Kröger, Shanmugaratnam, Scheib, et al., 2021; Scheib et al., 2014).

**FIGURE 1:**
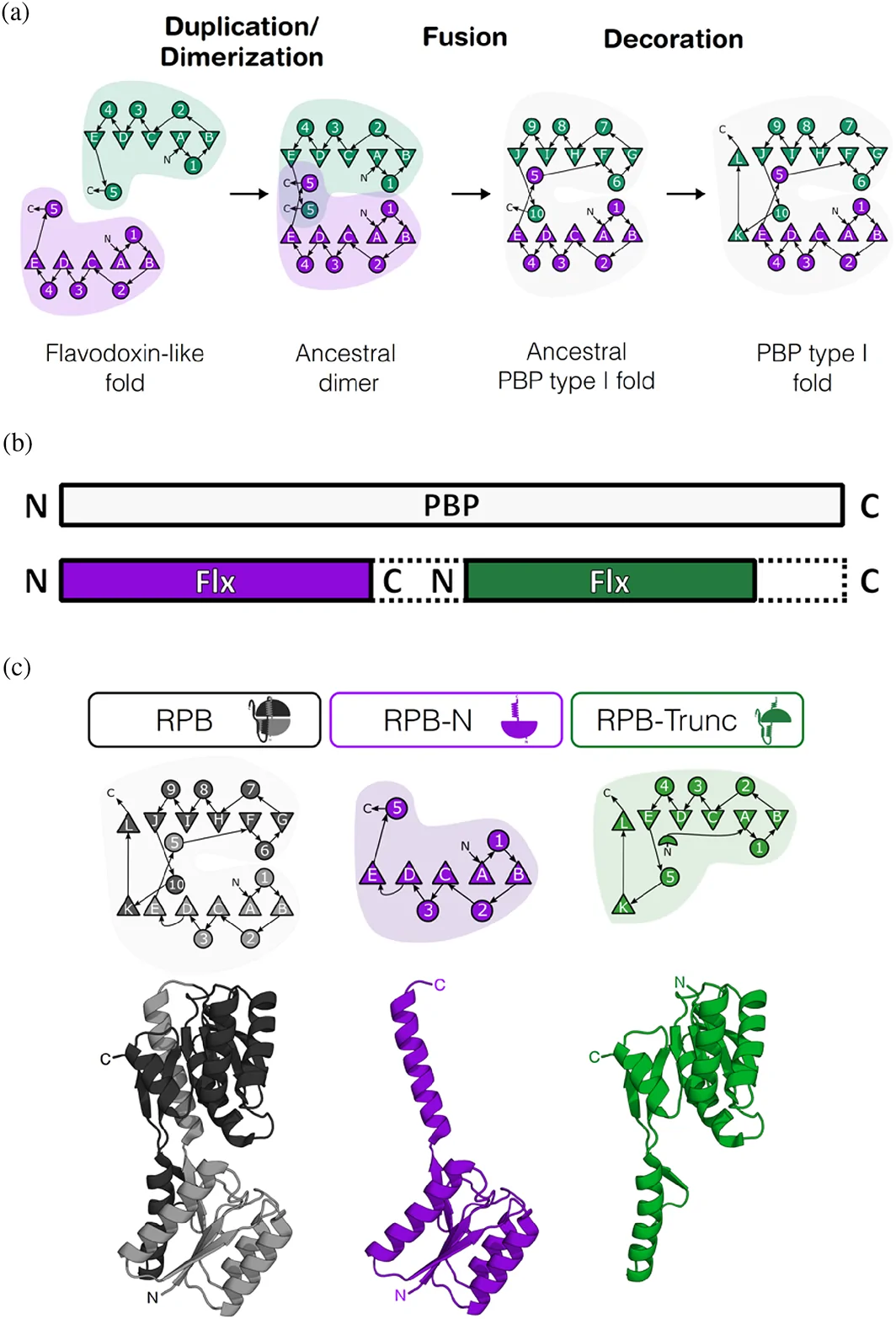
Proposed evolutionary trajectory of modern PBP‐type I proteins and the derived constructs used in this study. (a) Proposed steps that reconstruct the evolution of modern periplasmic‐binding‐protein (PBP) folds from an ancestral protein adapting the flavodoxin‐like fold (adapted from Fukami‐Kobayashi et al., 1999). A duplication and dimerization along with swaps in secondary structure led to the formation of an ancestral dimer. Subsequent fusion of the genes then led to the emergence of an ancestral PBP‐like fold and further changes of secondary structure to that of the modern PBP‐like type I fold. (b) Schematic representation of the profile‐profile alignments for a representative full‐length PBP with the flavodoxin‐like‐fold (Flx). (c) First‐generation constructs RBP (black), RBP‐N (violet), and RBP‐Trunc (green) were analyzed in this work to recreate the PBP‐halves.

Despite diversity in the sequences of different PBPs, a shared common ancestry has been proposed a while ago (Fukami‐Kobayashi et al., 1999; Louie, 1993). Their structural features, similarities in binding mechanism, and shared operon structure—with the PBP being on the same operon as the associated signaling proteins downstream—have long led to the theory that PBPs arose via gene duplication of a progenitor protein and subsequent diversification. However, it is unclear in which order these events might have occurred (Fukami‐Kobayashi et al., 1999). It has been previously suggested that this common ancestor could have been a CheY‐like protein adopting a flavodoxin‐like fold. Formation of an ancestral dimer in combination with a gene duplication and fusion event might have led to the typical bilobal structure of the modern PBP (Figure 1a), an event that has already been investigated for the evolution of other protein folds (Alvarez‐Carreño et al., 2022; Farías‐Rico et al., 2014; Toledo‐Patiño et al., 2019).

Although the sequences of modern PBPs have diversified from their evolutionary ancestors, the topology is predominantly conserved. There are mainly two classes of PBP, with a slight difference in the order of secondary structural elements. It is thought that the second class descends from already evolved class I PBPs even though sequence similarity is not high between the two folds (Fukami‐Kobayashi et al., 1999). They are in fact classified as independent folds of either PBP‐like I or PBP‐like II in SCOP (Chandonia et al., 2019), as being of the same topology level as flavodoxins (for type I) and an independent homology group (for type II) in ECOD (Cheng et al., 2015), and as two different superfamilies in CATH (Sillitoe et al., 2021). The application of modern bioinformatic resources has opened up new opportunities to revisit some of these concepts of evolutionary relationships, partially through emergence of tools to more efficiently probe sequence space also in the sub‐domain regime of proteins (Alva et al., 2015; Farías‐Rico et al., 2014; Ferruz et al., 2020; Nepomnyachiy et al., 2017).

In this work we combine the approach of a sequence profile‐profile comparison analysis using Hidden Markov Models (HMMs) with a structural comparison of the two lobes of the PBP‐like fold type I. Based on this analysis, the emergence of the PBP‐like fold via the duplication of a flavodoxin‐like ancestor can be revisited. To further substantiate the claim, we biophysically and structurally characterized truncated constructs of the ribose‐binding protein (RBP) from *Thermotoga maritima* that correspond to the proposed duplicated progenitor halves. We found that it is generally possible to obtain stable and well folded monomeric proteins expressing only the individual lobes of full‐length RBP. The two independent halves appear to readily form a heterodimer, while also reconstituting the ribose‐binding ability of the parental protein, with affinities in the same order of magnitude. These results suggest a plausible path for the evolution of modern PBPs and increase our understanding of the evolution of complex and multidomain proteins from smaller molecular components.

## 2 RESULTS AND DISCUSSION

### 2.1 Disassembling a modern RBP into likely progenitor halves

The proposed mechanism of a duplication event being responsible for the architecture of PBPs mostly relies on analysis of either the available structures of modern PBPs (Louie, 1993; Berntsson et al., 2010), or comparison of the sequences of PBP‐like and flavodoxin‐like proteins (Fukami‐Kobayashi et al., 1999). We wanted to investigate whether the duplication of the flavodoxin‐like progenitor is not only theoretically feasible, but also practically. To retrace the evolution of a PBP, we characterized constructs based on the halves of an RBP (Figure 1 and Table S1). This not only allows to probe the plausibility of this mechanism in general, but also offers an opportunity to investigate the individual impact of each subdomain‐part on the stability and function of modern PBPs.

We chose the RBP of *T. maritima* for this purpose. Not only does the thermophilic nature of this protein offer a robust system, but also a previously reported expression of a 21 kDa truncated version (Cuneo et al., 2008) made this an excellent candidate for a model system. To generate an overview of possible intersections, a multiple sequence alignment with RBP as input was generated with HHpred (Figure S1). The results show not only the alignment of other full‐length PBPs on the query sequence but also an alignment of the individual lobes. The lobes align with a clear cut being observable between residues 30–155 and 156–310 of the RBP (numbering consistent with Uniprot entry Q9X053). To compare this with the alignment of the proposed progenitor flavodoxin‐like proteins, the same alignment was generated within the *Fuzzle* database (Ferruz et al., 2021), which automatically excludes sequences of the same fold. It shows that flavodoxin‐like proteins align with both the corresponding N‐ and C‐terminal halves of the PBP sequence (Ferruz et al., 2021). While alignment of flavodoxin‐like proteins with RBP seems to heavily favor hits on the N‐terminal half, some hits are also found with the C‐terminal half. A reason why less hits might be observed on the C‐terminal half of this modern RBP could be a result of the duplication and a subsequent decoupling of the sequences of the two halves, resulting in increased divergence from the progenitor flavodoxin‐like protein, and thereby making it harder to identify.

While the existence of the earlier reported truncated RBP variant could be an artifact of the expression in *Escherichia coli* (Cuneo et al., 2008), it is also possible to be a natural occurrence. A shortened version of a solute‐binding protein with a proposed biological function has been reported previously (Bae et al., 2018). Although it is unclear why these single‐lobed proteins might exist, the truncated RBP could also carry biological significance. Thus, we chose to use the truncated protein that is roughly the equivalent of the single‐lobed half as a base for the constructs used in this study.

For the first generation of constructs we took to the lab, the sequence of the full‐length RBP was disassembled into the corresponding halves (Figure 1 and Table S1). The site of dissection was determined by structural alignment of RBP in absence of ribose (PDB ID: 2FN9) to the top‐scoring flavodoxin‐like proteins in the HHpred analysis, resulting in the constructs RBP‐N (amino acid 30–153 of RBP) and RBP‐C (amino acid 157–291) that contain a sequence identity and similarity to each other of 16.8% and 25.6%, respectively. These constructs were expressed and characterized using biochemical and biophysical methods.

### 2.2 RBP halves are well folded

Upon overexpression of the RBP halves in *E. coli* the protein RBP‐N was found in the soluble fraction of the cell extract while RBP‐C was located in inclusion bodies. Since full‐length RBP also features a C‐terminal decoration common to modern PBPs which does not correspond to any elements in the canonical flavodoxin‐like architecture, the additional elements (two β‐strands that facilitate another cross‐over between the two lobes and extend the central β‐sheet of the two halves) had been removed in RBP‐C. This removal might be the reason why in contrast to RBP‐N, which expressed solubly, could be purified to homogeneity, and remained stable at concentrations above 15 mg mL^−1^, RBP‐C only expressed insolubly. We therefore decided to continue the investigation with the truncated construct RBP‐Trunc (residues 142–310) instead (Figure 1c), which is related to the RBP‐C half and expressed solubly with similar stability to the N‐terminal construct RBP‐N.

**FIGURE 2:**
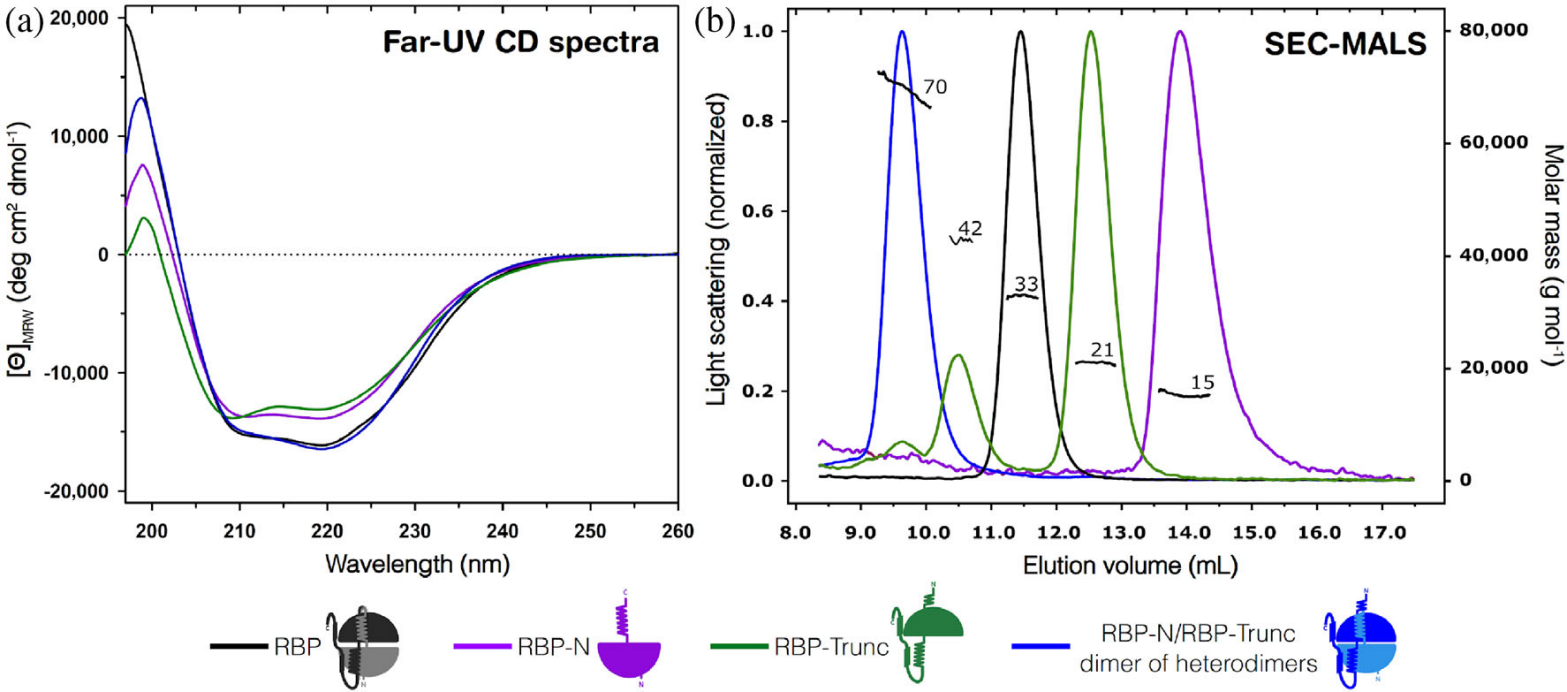
Biophysical characterization of the first‐generation constructs. (a) Far‐UV CD spectra at 20°C collected in 10 mM sodium phosphate, 50 mM sodium chloride, pH 7.8. (b) SEC‐MALS experiments performed in 10 mM sodium phosphate, 50 mM sodium chloride, 0.02% sodium azide, and pH 7.8. Numbers indicate the molecular weight determined after data analysis. Values derived from the experiments are reported in Table S2. In both panels, the color code is RBP (black), RBP‐N (violet), RBP‐Trunc (green), and the RBP‐N/RBP‐Trunc dimer of heterodimers (blue).

**FIGURE 3:**
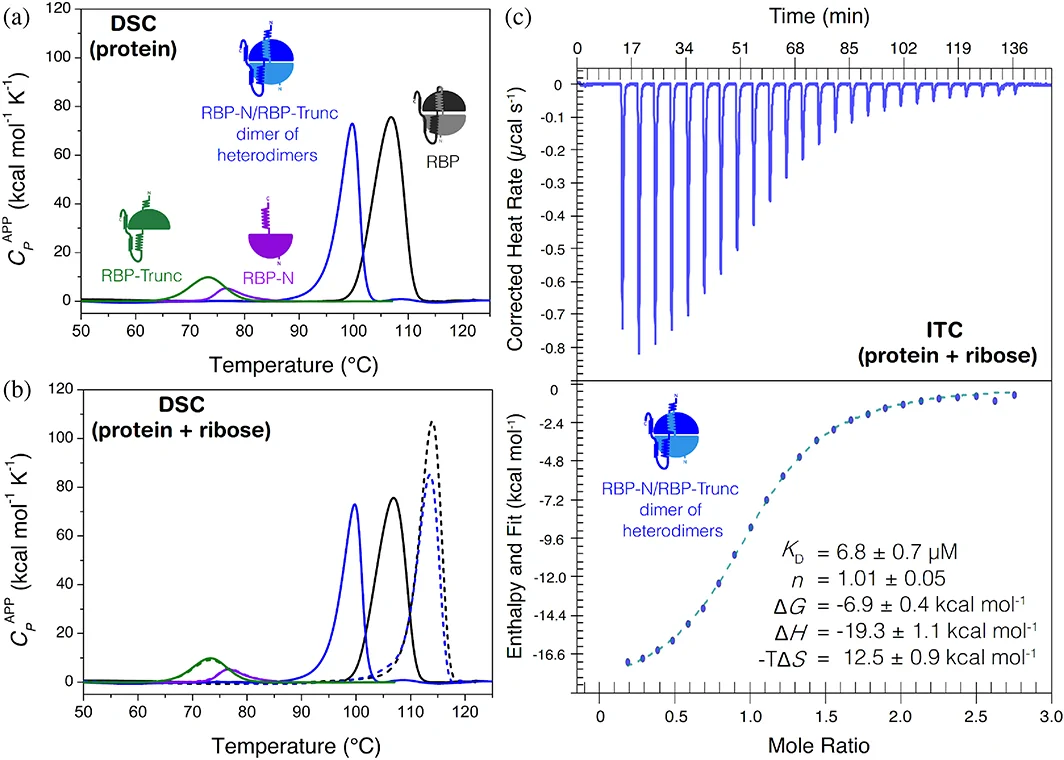
Thermodynamic characterization of the first‐generation constructs and their interaction with ribose. (a) DSC endotherms at 1.5°C min^−1^ of the halves RBP‐Trunc (green), RBP‐N (violet), the RBP‐N/RBP‐Trunc dimer of heterodimers (blue), and the full‐length RBP (black) without ribose and (b) with 0.5 mM ribose. Experiments were performed in 10 mM sodium phosphate, 50 mM sodium chloride, pH 7.8, and the physical and chemical baselines have been subtracted. (c) Representative ITC measurement for ribose binding of the RBP‐N/RBP‐Trunc heterodimer. Baseline‐subtracted raw data are shown at the top while the binding isotherms (blue circles) fitted to a 1:1 model (dotted line) are presented at the bottom. ± at the reported parameters indicate the standard deviation of 3 independent experiments. Titrations were performed at 20°C in 10 mM sodium phosphate, 50 mM sodium chloride, pH 7.8.

Both RBP‐N and RBP‐Trunc display far‐UV CD spectra with the signature ɑ‐helix minima at 208 and 222 nm and moderated by the signal of the β‐sheet at 218 nm, both characteristic for α/β‐proteins (Figure 2a) and comparable with the native full‐length RBP. Comparison of the intrinsic fluorescence (IF) also corroborates this (Figure S2A), indicating that the constructs are well folded since the intensity maximum suggests that the aromatic residues are buried from the solvent. In addition, DSC endotherms show cooperative thermal‐unfolding transitions with melting temperatures and enthalpy values close to full‐length RBP (Table 1, Table S3, and Figure 3), confirming the characteristics of well‐folded proteins (see next section for further details).

**TABLE 1:** Characterization summary (oligomeric state and thermostability with/without ribose) for the constructs analyzed in this work.

Protein			Oligomeric state	*T*_m_ (°C) (protein)	*T*_m_ (°C) (protein + ribose)	Ribose binding^a^
First generation	RBP	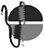	Monomer	106.9 ± 0.4	114.0 ± 0.9	Yes
	RBP‐N	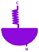	Monomer	76.6 ± 0.2	76.7 ± 0.3	No
	RBP‐Trunc	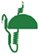	Monomer (90%) Homodimer (10%)	73.3 ± 0.1	73.4 ± 0.2	No
	RBP‐N/RBP‐Trunc mixed heterodimer	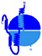	Dimer of heterodimers	99.7 ± 0.3	113.5 ± 0.4	Yes
Second generation	RBP‐N_N‐His_	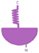	Monomer	73.2 ± 0.1	73.1 ± 0.2	No
	RBP‐TruncII_N‐Strep_	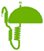	Monomer	70.6 ± 0.2	70.8 ± 0.3	No
	RBP‐TruncII_N‐His_	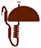	Monomer	70.4 ± 0.4	70.9 ± 0.5	No
	RBP‐N_N‐His_/RBP‐TruncII_N‐Strep_ co‐expressed heterodimer	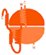	Heterodimer	104.8 ± 0.3	113.9 ± 0.4	Yes
	RBP‐N_N‐His_/RBP‐C_N‐Strep_ co‐expressed heterodimer	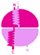	n.d.	68.4 ± 0.5	83.5 ± 0.9	Yes

^a^ Interaction with ribose was determined by changes in thermostability (*T*_m_) and enthalpy (Δ*H*) parameters comparing DSC endotherms collected without and with 0.5 mM ribose.

Further analysis with SEC‐MALS (Figure 2b) confirmed the monomeric state of RPB and RBP‐N. However, RBP‐Trunc is in an equilibrium of mostly monomeric species and homodimers, with higher oligomers also being present (Table S2). These results indicate that the RBP halves are well folded proteins and express mainly as monomeric systems, similar to those observed in another PBP, HisJ (Chu et al., 2013). To follow up on this, we continued to study their properties in the presence of each other.

### 2.3 RBP halves form a heterodimer whose structure is identical to full‐length RBP

Since one of the steps proposed in the evolution of the modern PBP architecture involves an ancient dimer, we investigated whether the obtained constructs had the ability to reconstitute the full‐length RBP fold. For this, the individually purified RBP‐N and RBP‐Trunc were mixed in an equimolar ratio and then analyzed. The far‐UV CD spectra (Figure 2a) show a significant change of the signal to the individual constructs, with the signal of the mixed RBP‐N/RBP‐Trunc resembling that of the full‐length RBP. A similar behavior can be observed in the IF spectra (Figure S2A), where the original characteristics of the full‐length protein are reconstituted when mixed *in vitro*, hinting at the formation of an RBP‐N/RBP‐Trunc heterodimer. Complex formation is supported by SEC‐MALS analysis where only one well‐defined peak is displayed corresponding to the mass of the RBN‐N/RBP‐Trunc dimer of heterodimers (Figure 2b and Table S2).

Additionally, DSC analysis of the proteins supports the formation of a heterodimer that resembles the parental protein. All endotherms show clear single and cooperative transitions, as has been observed for other PBPs such as maltose‐, arabinose‐, and histidine‐ binding proteins (Fukada et al., 1983; Ganesh et al., 1997; Kreimer et al., 2000). However, RBP and its halves showed irreversible thermal unfolding possibly due to their thermophilic nature, contrary to most PBPs which exhibit reversible transitions (Aggarwal et al., 2011; Fukada et al., 1983; Ganesh et al., 1997; Kreimer et al., 2000; Prajapati et al., 2007; Vergara et al., 2020). While full‐length RBP has a *T*_m_ of 106.9°C similar to the one previously reported for the construct (Cuneo et al., 2008), RBP‐N and RBP‐Trunc show lower thermostability with a *T*_m_ of 76.6 and 73.3°C, respectively (Figure 3a, Table 1, and Table S3). The results show that the halves have native‐like properties, that interdomain interactions are important in RBP and that these provide relevant stabilization, in the same way as has been described for other multidomain proteins (Brandts et al., 1989; Careaga et al., 1995; Kantaev et al., 2018; Liu et al., 2019; Vergara et al., 2023; Vogel et al., 2004; Wenk et al., 1998). This decrease in thermostability of the individual constructs is compensated by the formation of the RBP‐N/RBP‐Trunc heterodimer, whose *T*_m_ is shifted by more than 20–99.7°C, more closely resembling that of RBP.

The same tendency is observed when comparing the changes in Δ*H* of the individual and mixed constructs (Table S3), with a considerable increase of 240 kcal mol^−1^ in the unfolding enthalpy, which is significantly higher than only the sum of the individual halves (115 kcal mol^−1^). These differences indicate that more accessible surface area is exposed upon unfolding, which is most likely due to the formation of an extensive interface and interdomain interactions important for protein stability and function as present in RBP, confirming the interaction between RBP‐N and RBP‐Trunc. These results exhibit a similar behavior as observed in the lysine‐arginine‐ornithine (LAO) binding protein (Vergara et al., 2023) but differ from those of a previous study of the type‐II PBP protein HisJ (Chu et al., 2013) where the isolated lobes do not interact with each other in the presence or absence of histidine, suggesting that in HisJ only one lobe is important for ligand binding and the other is considered to play a supporting role in the dynamics of binding and in protein stability.

The differences in *T*_m_ and Δ*H* of the native proteins and the mixed heterodimer can be explained by the carry‐over of ribose from the purification. It is notoriously hard to remove bound ligands from the expression medium when purifying solute‐binding proteins that have a high affinity for their ligands (Structural Genomics Consortium et al., 2008). Due to its high stability and irreversible thermal unfolding, RBP resisted all attempts of refolding, making purification of a sample removed of all residual ribose not possible, and for this reason always some ribose was carried‐over in the purified RBP, increasing the measured *T*_m_ and Δ*H* by a ligand stabilization mechanism. Since the individual halves of RBP do not show any binding of ribose (Figure S3), carry‐over is not expected to occur during purification, therefore no additional stabilizing effect of ribose binding is expected.

**FIGURE 4:**
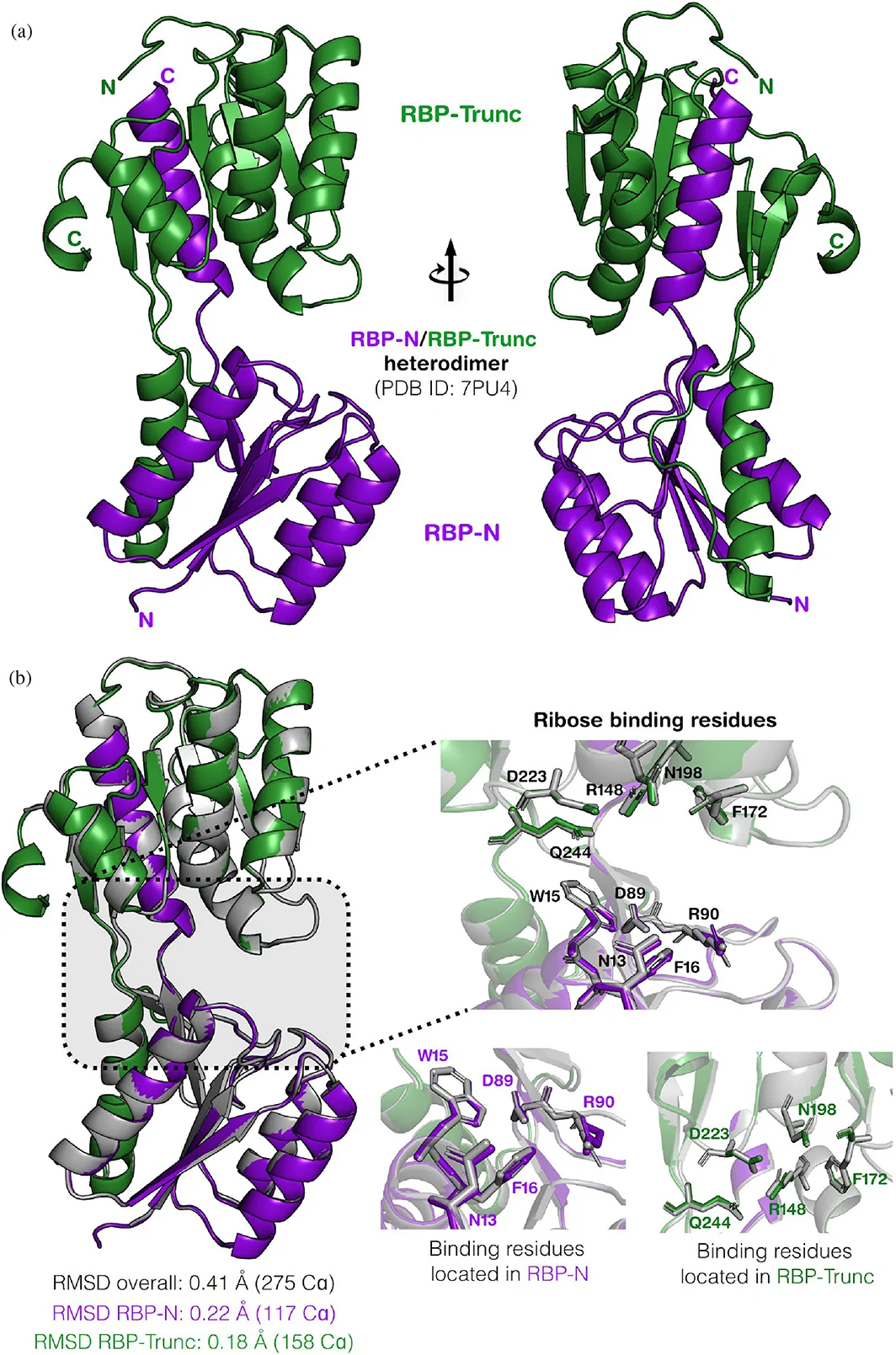
Crystal structure of the RBP‐N/RBP‐Trunc heterodimer in unliganded conformation. (a) Cartoon representation of RBP‐N (violet) and RBP‐Trunc (green) heterodimer (PDB ID: 7PU4) forming a native‐like conformation as full‐length RBP. (b) Structural comparison of RBP‐N/RBP‐Trunc heterodimer and RBP (PDB ID: 2FN9; gray). RMSD values are reported for the entire heterodimer, and halves RBP‐N and RBP‐Trunc. Inset shows the ribose binding residues in the full structure (top) and separated in each half (bottom); numbering is based on the RBP sequence.

Next, we determined the crystal structure of the RBP‐N/RBP‐Trunc heterodimer (PDB ID: 7PU4) (Figure 4 and Table S4). The two halves indeed reconstitute the canonical RBP fold with high structural similarity, showing a Cɑ‐RMSD of 0.41 Å of the heterodimer to the previously reported structure of unliganded RBP (PDB ID: 2FN9), confirming the aforementioned spectroscopic and calorimetric results. The heterodimer displays the same opening and twisting angle as the paternal protein, an important indicator of a native‐like configuration of the heterodimer. The asymmetric unit of the crystal structure shows a dimer of RBP‐N/RBP‐Trunc heterodimers (Figure S5), which is in agreement with the oligomeric state observed in SEC‐MALS experiments (Figure 2); however, further analysis is needed to determine the precise conformation of the dimer of heterodimers in solution. The observed heterodimer interface in the asymmetric unit is mostly related to the interaction of C‐terminal residues of RBP‐Trunc located in the hinge region and their corresponding ones in the crystallography mates, ruling out the possibility that dimerization results from the extra elements left out in RPB‐Trunc. Finally, a closer look at the side‐chains involved in ribose binding reveals an almost identical orientation compared to the unliganded state of the native RBP, suggesting the correct formation of the preformed binding site (Figure 4b). Since all these results showed that the separately purified RBP halves can reassemble the structural conformation of full‐length RBP *in vitro,* we next wanted to determine whether this RBP‐N/RBP‐Trunc heterodimer is also a functional RBP protein.

### 2.4 The reassembled heterodimer binds ribose with a comparable affinity to full‐length RBP

The structural similarity of the heterodimer with the full‐length RBP suggests that also the ribose binding function might be reconstituted. To investigate this, we first analyzed by DSC if ribose binding increases protein thermostability. Specific protein‐ligand interaction commonly causes an increase in protein thermostability, which is due to the coupling between binding and unfolding processes under thermodynamic equilibrium (Cooper et al., 2000; Privalov, 1979).

The isolated RBP‐N and RBP‐Trunc do not show any sign of stabilization upon addition of ligand (Figure S3). This differs from type II PBP‐like fold proteins such as LAO, ArgBP, or HisJ, in which it has been shown that albeit with lower affinity, one of the isolated lobes is able to bind its respective ligand (Chu et al., 2013; Smaldone et al., 2020; Vergara et al., 2023).

In type I PBP‐like fold proteins like RBP, the binding residues are distributed almost equally between the two lobes, while in many type II PBPs almost all binding residues are present only in one lobe (mostly in the discontinuous one). In addition, changes in the hinge connections between the distinct types of PBPs also modify the binding properties and dynamics (Bermejo et al., 2009, 2010; Chu et al., 2013; Gouridis et al., 2021; Ortega et al., 2012; Pistolesi et al., 2011). These differences in the architecture of type I and II PBP‐like fold proteins could explain why one of the isolated lobes from type II proteins such as LAO, HisJ, and ArgBP is able to bind their respective ligand while none of the individual lobes from type I PBPs have been shown to be competent by themselves. Variations in ligand affinity and promiscuity for some of the studied PBPs (Chu et al., 2013; Kröger, Shanmugaratnam, Ferruz, et al., 2021; Kröger, Shanmugaratnam, Scheib, et al., 2021; Vergara et al., 2020) indicate that possibly the PBP ancestor was able to bind some ligands but with considerably lower affinity, similarly to what has been reported for enzyme evolution (Copley, 2020; Khersonsky & Tawfik, 2010; Tawfik, 2020). In a plausible scenario, after duplication and fusion of the flavodoxin‐like fold ancestor (Figure 1a), type I PBP‐like fold proteins were able to evolve obtaining increased selectivity and affinity for specific compounds but still sharing almost equally the ligand binding residues between both domains, as has been observed for RBP.

In contrast to the isolated RBP domains, an increase in *T*_m_ can be observed upon addition of 0.5 mM ribose (Figure 3b) to the RBP‐N/RBP‐Trunc heterodimer, with the amplitude of the absorbed heat changes being dependent on ligand concentration. The *T*_m_ of the ligand‐bound RBP‐N/RBP‐Trunc heterodimer increases by almost 14°C from 99.7 to 113.5°C, comparable to the stabilization of ligand‐bound RBP by around 7°C to 114.0°C (Figure S3 and Table S3) and similar to the one observed in other PBPs when binding their respective high‐affinity ligands (Fukada et al., 1983; Ganesh et al., 1997; Kreimer et al., 2000).

In addition, an increase of 129 kcal mol^−1^ was observed in the unfolding Δ*H* for the ligand‐bound RBP‐N/RBP‐Trunc heterodimer in comparison to the unbound form, deducing that large‐scale rearrangements in the solvent‐exposed surface in the heterodimer accompanies ligand binding, thereby confirming a functional protein that behaves similar to full‐length RBP. The greater amount of thermostabilization in the heterodimer in comparison to RBP can again be explained by residual ribose carried over in the purification of RBP already stabilizing the protein. However, at the same concentration of ribose the level of stabilization of the heterodimer is almost identical to that of RBP, with the heterodimer displaying a native‐like thermostability. Interestingly, the significant increase in stability can also be observed when adding ribose to a non‐native SDS‐PAGE. At concentration of 1 mM ribose or higher, a dimer (and higher oligomers) can be detected, indicating that the addition of SDS and the subsequent heating to 99°C is not enough to dissociate the ribose‐bound stabilized heterodimer (Figure S4).

Additionally, to DSC analysis, ribose binding of the RBP‐N/RBP‐Trunc dimer was determined by ITC. Ribose‐binding isotherms (Figure 3c) showed a sigmoidal profile with the ribose binding constant (*K*_D_ = 6.8 ± 0.7 μM) in a concentration range comparable to other previously studied solute‐binding proteins (Schreier et al., 2009), implying that the binding of ribose can be regained after *in vitro* mixing the previously dissected RBP halves. In fact, ligand affinity is not significantly affected by the assembly. Now the question remained, whether this reassembled functional heterodimer can also be formed *in vivo* upon co‐expression of both halves.

### 2.5 RBP halves form a functional heterodimer when co‐expressed in *E. coli*

**FIGURE 5:**
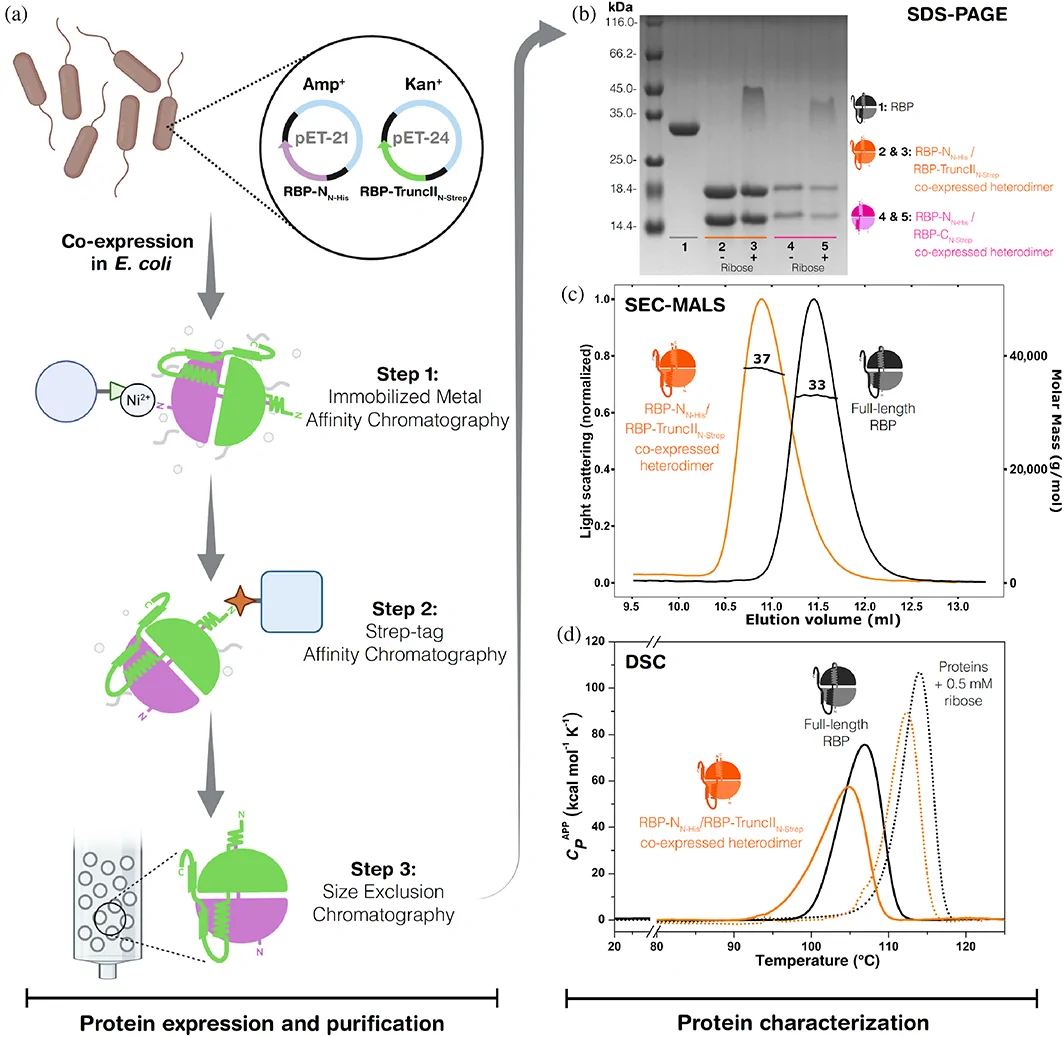
Co‐expression in *Escherichia coli* and characterization of the second‐generation heterodimers. (a) Schematic workflow of the co‐expression beginning with the transformation of *E. coli* with the two plasmids carrying RBP‐N_N‐His_ and RBP‐TruncII_N‐Strep_. Subsequent alternating affinity chromatographies utilizing two different tags assure purification of only the RBP‐N_N‐His_/RBP‐TruncII_N‐Strep_ heterodimer, followed by a final size exclusion step. **(**b) SDS‐PAGE showing the co‐purified heterodimers. RBP (lane 1), co‐expressed RBP‐N_N‐His_/RBP‐TruncII_N‐Strep_ heterodimer without ribose (lane 2) and with 0.5 mM ribose (lane 3), co‐expressed RBP‐N_N‐His_/RBP‐C_N‐Strep_ heterodimer without ribose (lane 4) and with 0.5 mM ribose (lane 5). (c) SEC‐MALS measurements of RBP‐N_N‐His_/RBP‐TruncII_N‐Strep_ heterodimer (orange line) in comparison with full‐length RBP (black line). Numbers indicate the determined experimental molecular weight. (d) DSC endotherms of RBP‐N_N‐His_/RBP‐TruncII_N‐Strep_ heterodimer and RBP in absence (continuous lines) and presence of 0.5 mM ribose (dotted lines).

To investigate whether the heterodimer of RBP‐N and RBP‐Trunc already forms during the expression in *E. coli*, a second generation of constructs was created (Table S1). To ensure that at least one plasmid copy of each construct stays in each cell, the coding sequences were assembled in a vector imparting resistance to either ampicillin or kanamycin, respectively. Since there was no control of expression levels and we wanted to only obtain heterodimer in the subsequent purification, we opted for adding two different affinity tags to each construct (Figure 5a). The resulting constructs are RBP‐N_N‐His_ and RBP‐TruncII_N‐Strep_ (Table S1) with affinity labels located at the N‐terminus. By utilizing a three‐step purification approach using the different affinity tags on each protein half and a subsequent SEC step for polishing, we can assure that only already formed heterodimers are retained as confirmed by the SDS‐PAGE showing a band at the corresponding sizes of both RBP‐N_N‐His_ and RBP‐TruncII_N‐Strep_ and thermal resistance upon addition of ribose (Figure 5b). Similar to the behavior of the 1st generation constructs, the far‐UV CD and fluorescence spectra showed a reconstitution of characteristics almost identical to the native RBP (Figure S2B and Figure S6A). The molecular weight determined by SEC‐MALS also corresponds to the heterodimer (expected mass: 36.8 kDa/determined mass: 37.3 kDa), with no higher oligomers present (Figure 5c and Table S2).

Similar to the mixed RBP‐N/RBP‐Trunc heterodimer, the co‐expressed and co‐purified heterodimer shows an increase in thermostability in the presence of ribose (Figure 5d and Table 1), indicating a functional heterodimer. The *T*_m_ of RBP‐N_N‐His_/RBP‐TruncII_N‐Strep_ increases by 9.1°C (from 104.8 to 113.9°C) after addition of 0.5 mM ribose, showing a similar trend of stabilization as the full‐length RBP (Figure S3 and Table S3) and also to the 1st generation of halves. In view of the heterodimer being formed in the cells during co‐expression, the same behavior of carrying over residual ribose from *E. coli* is expected to increase the measurable *T*_m_ of the RBP‐N_N‐His_/RBP‐TruncII_N‐Strep_ heterodimer.

Since the formation of the heterodimer appears to stabilize the individual protein halves and yields properties almost identical to full‐length RBP, we set out to retry the expression of the previously insolubly expressing RBP‐C in the hopes that the co‐expression and formation of the heterodimer *in‐cell* could rescue the protein. The second generation RBP‐C_N‐Strep_ was purified along RBP‐N_N‐His_ analogously to the previous co‐expression assay (Figure 5a). Interestingly, we were able to obtain a small amount of purified heterodimer after the affinity chromatography and subsequent SEC (Figure 5b), with a high‐oligomer band still being visible in the SDS‐PAGE after addition of ribose, which indicates retention of binding function. This characteristic is confirmed by DSC measurements of the heterodimer with and without ribose (Figure S7). While the overall transition is massively decreased for the unbound proteins (*T*_m_ = 68.4°C for RBP‐N_N‐His_/RBP‐C_N‐His_ co‐expressed heterodimer versus *T*_m_ = 106.9°C for full‐length RBP), the strong stabilization after addition of ribose is still observed (15.1°C of *T*_m_ increase to 83.5°C). The total shift is comparable with that in RBP, albeit with some fraction of the protein still appearing to be in a ligand‐free state (Figure S7), indicating a possible reduction in ribose binding affinity or different populations of the purified heterodimer. The ability of RBP‐N_N‐His_ to recover not just the soluble expression of RBP‐C_N‐Strep_ via the formation of the heterodimer, but also the heterodimer to retain its function, showcases the inherent versatility of this fold and gives insights into its evolution.

The PBP architecture, like multidomain proteins, illustrates how the modular reuse of domains can generate more complex macromolecules, that often include the addition of extra secondary structural elements or even larger decorations towards acquiring new functions (Das et al., 2015; Ferruz et al., 2020; Gouridis et al., 2021). In a global manner, these changes have shown how domain‐domain interactions, previously not present in the single independent units, are essential for the folding, stability, and function of multidomain proteins, especially for those residues located close to the interdomain interface (Vogel et al., 2004) and for modulation of binding‐site solvation (Vergara et al., 2020; Vergara et al., 2023). Stabilizing interdomain interactions are useful to avoid misfolding and aggregation in multidomain proteins (Han et al., 2007) and moreover, domain‐domain interactions can control the dynamics and kinetics between open and closed states, being critical factors for the transport rate of PBPs (Gouridis et al., 2015). This suggests that after the duplication and fusion of an ancestral protein that corresponded to an individual RBP lobe, the entire protein sequence now works as an integrated functional unit, where folding, stability, and binding function are interlinked. This allows the protein to evolve new properties such as gaining ligand selectivity, increasing binding affinity, and modifying the dynamics of ligand binding and transport by including open and closed states. These significant closing and twisting motions observed in PBPs (Chu et al., 2013; Gouridis et al., 2021; Kröger, Shanmugaratnam, Ferruz, et al., 2021; Vergara et al., 2020) would not be possible without the evolution of RBP as a single functional unit.

## 3 CONCLUSIONS

### 3.1 Implications for the evolution of the PBP fold and protein engineering approaches

The data presented here shows how a modern PBP can be disassembled into its two lobes, and how when they are combined *in vitro* or *in vivo* the formed dimer is able to perform its original function. The individual parts readily assemble to form a heterodimer, not just when mixing the individually purified lobes, but also within the cell upon co‐expression. While the N‐ and C‐terminal lobes appear to be stable and well‐behaved proteins on their own, formation of the heterodimer almost completely restores the characteristics of the full‐length RBP, confirming the importance of interdomain interactions on the evolution, stability and function of the PBP fold, similarly to what has been reported for other multidomain proteins (Alvarez‐Carreño et al., 2022; Han et al., 2007; Vogel et al., 2004).

Analysis of the stability and binding abilities indicate native‐like properties, and the crystal structure of the heterodimer being nearly identical to that reported for RBP supports this conclusion. This versatility of the PBP fold can be explained by the inherent malleability of proteins of the flavodoxin‐like (and related) folds. Several structures with swapped elements have been reported for flavodoxin‐like proteins (e.g., PDBs: 4Q37, 6ER7/6EXR, 3C85; Paithankar et al., 2019; Farías‐Rico et al., 2014) as well as TIM‐barrel proteins (PDB 6QKY; Michalska et al., 2020), which are also thought to be related to the flavodoxin‐like fold (Romero‐Romero et al., 2021). Further, we had previously observed swapped elements in circular‐permuted constructs of RBP (PDBs: 7QSP, 7QSQ; Michel et al., 2023). This tendency of the structural archetype to enable formation of swapped elements could have been an important characteristic promoting the emergence of the ancestral dimer thought to be the progenitor of modern PBPs. While the two halves we describe in this work are derived from an already evolved protein, they could still be seen as a vestige of this ancestral dimer. Interestingly, the crystal structure of a flavodoxin‐like fold protein with an identical arrangement of secondary structure elements has been described already, albeit it is unclear whether the observed structure is an artifact of the non‐physiological crystallographic conditions (Lewis et al., 2000).

Since the heterodimer corresponds to the proposed ancestral dimer in the evolutionary trajectory (Figure 1a) while still retaining function with native‐like properties, this presents new insight into the mechanisms behind such a duplication event. Not only does the orientation of the two lobes create the binding cleft characteristic for PBP‐like proteins, but also the general restraints on the movement of the lobes lower the entropic cost of ligand binding. Our findings showcase the feasibility of a functional heterodimer similar to the proposed ancestral one to also assemble within cells, giving way to the argument that the duplication and fusion of the progenitor flavodoxin‐like protein might have happened independent of the gain of function, indicating no evolutive pressure on single domains but on the full‐length RBP.

Adopting this approach and expanding it to incorporate a diverse set of functions could also be used for protein engineering purposes. This is traditionally done by inserting a domain for readout into the sequence of an existing PBP, with the optimal placement of the insertion sites being one of the major challenges (Ribeiro et al., 2019; Tullman et al., 2016). Further studies will have to show that the retracing of the duplication is applicable for other PBPs as well, but one could imagine its usage in creating modular switch systems not just *in vitro*, but also *in vivo*.

## 4 MATERIALS AND METHODS

### 4.1 Reagents and solutions

Analytical grade chemicals were used for all the experiments. Water was distilled and deionized.

### 4.2 Identification of the protein halves and sequence analysis

The bioinformatic analysis to trace the sequence similarities between the RBP and flavodoxin‐like proteins was done using the HHpred server which is part of the HHsuite (Gabler et al., 2020) (Figure S1). The sequence of full‐length RBP (UniProt‐ID: Q9X053) excluding the extracellular transport signal was run with standard parameters, but disabling secondary structure scoring and increasing the number of maximal hits to 10,000 to also obtain sequences with lower probability scores. Based on the alignment of both the other PBP lobes and the hits with the flavodoxin‐like proteins, the cutting points were determined at position 30–155 for RBP‐N, 142–310 for RBP‐Trunc, 156–310 for RBP‐TruncII, and 157–291 for RBP‐C (Table S1).

### 4.3 Cloning and generation of RBP‐constructs

The gene fragment for wild‐type RBP lacking the periplasmic signal sequence as well as the primers used for assembly were provided by Eurofins Genomics. To generate the gene fragments for RBP‐N and RBP‐Trunc, a polymerase chain‐reaction with the corresponding primer was conducted with the full sequence as template. Additionally, a QuikChange® site‐directed mutagenesis was performed to obtain the M142A mutation of the full‐length RBP to prevent the translation of the truncated protein (henceforth called RBP). The fragment of full length RBP was cloned into empty pET‐21 using the *Nde*I/*Xho*I restriction sites. Analogously generated fragments for RBP, RBP‐N, and RBP‐Trunc were all subsequently cloned using T5 exonuclease‐dependent assembly (Xia et al., 2019). All constructs were verified by sequencing.

Gene synthesis and cloning for the co‐expression assay were provided by Biocat. The differently tagged constructs of RBP‐TruncII and RBP‐N_N‐His_ were cloned into pET24‐ and pET21‐vectors, respectively. Individual clones were obtained by transforming *E. coli* BL21 (DE3) cells by adding 50 ng of purified plasmid, heat shock and subsequent plating on agar‐plates supplemented with the corresponding antibiotic. To obtain cells carrying the two different plasmids needed for the co‐expression assay, 50 ng of each plasmid were added to the *E. coli* BL21 (DE3) cells, heat shocked and then grown on plates containing the two selecting antibiotics.

### 4.4 Expression and purification of RBP‐constructs

The transformant *E. coli* BL21(DE3) were grown in *Terrific broth* media (TB) at 37°C to an OD_600_ of 1.2 in the presence of the corresponding antibiotics (ampicillin 100 μg mL^−1^; kanamycin 50 μg mL^−1^). Protein expression was induced by the addition of Isopropyl‐β‐thiogalactopyranoside to a concentration of 1 mM and a total time of 18 h at 20°C. Cells were harvested via centrifugation (5000 × G, 15 min), resuspended in the corresponding binding buffer (20 mL g^−1^ wet weight), lysed by sonication and subsequently centrifuged to remove remaining cell debris (40,000 × G, 1 h). The cleared lysate was filtered through a 0.22 μm filter previous to the affinity column step.

For the constructs carrying a hexahistidine affinity tag, Immobilized Metal Ion Chromatography (IMAC) was performed on a Cytiva HisTrap 5 mL column equilibrated with buffer (20 mM MOPS, 500 mM sodium chloride, 10 mM imidazole, pH 7.8). Elution was performed with a step of IMAC‐Elution‐Buffer (20 mM MOPS, 500 mM sodium chloride, 600 mM imidazole, pH 7.8) at 40%, and fractions corresponding to the eluted protein pooled and concentrated to a volume suitable for the size exclusion chromatography (SEC) step.

Strep‐Tactin affinity chromatography was used for constructs with a StrepII‐Tag, which were loaded onto a Cytiva StrepTrap HP 5 mL column equilibrated with Strep‐Trap binding Buffer (100 mM Tris–HCl, 150 mM sodium chloride, 1 mM EDTA, pH 7.8) and eluted with Strep‐Trap elution Buffer (100 mM Tris–HCl, 150 mM sodium chloride, 1 mM EDTA, 2.5 mM Desthiobiotin, pH 7.8), pooled and concentrated analogous to the IMAC purification. To facilitate purification of the individual constructs, the Strep‐Tag of RBP‐Trunc_N‐Strep_ was switched to a His_6_‐Tag, creating RBP‐Trunc_N‐His_.

For the purification of the co‐expressed constructs, to assure survival of cells carrying only the two plasmids, the LB medium used for the production was supplemented with both Ampicillin and Kanamycin (100 and 50 μg mL^−1^, respectively). Cell lysis was performed as with the individual constructs, and the lysate first loaded on the HisTrap column. The eluted fractions corresponding to the tagged protein were pooled and applied onto a StrepTrap column. Similarly, eluted fractions were pooled and concentrated to a volume suitable for application onto the Superdex column.

SEC was performed as final purification step for all constructs on a Cytiva Superdex 26/600 75 pg with an isocratic elution using buffer 10 mM sodium phosphate, 50 mM sodium chloride, pH 7.8. Fractions consistent with the proteins of interest were analyzed by SDS‐PAGE, pooled, flash frozen in liquid nitrogen, and stored at −20°C until further analysis.

### 4.5 Far‐UV circular dichroism

Far‐UV circular dichroism (CD) measurements were performed at 20°C in buffer 10 mM sodium phosphate, 50 mM sodium chloride, pH 7.8 in a Jasco J‐710 spectropolarimeter equipped with a Peltier device to control temperature (PTC‐348 WI). Spectra were collected using 5 μM protein concentration for RBP and the heterodimers, and 10 μM for the other constructs in a 2 mm cuvette, 195–260 nm wavelength range, and 1 nm bandwidth. After buffer subtraction, raw data were converted to mean residue molar ellipticity ([*Θ*]) with [*Θ*] = *Θ/l C N*_*r*_, where *Θ* is the ellipticity signal in millidegrees, *l* is the cell path in mm, *C* is the molar protein concentration, and *N*_r_ is the number of amino acids per protein (Greenfield, 2006).

### 4.6 Intrinsic fluorescence

Intrinsic fluorescence (IF) spectra were collected on a Jasco FP‐6500 spectrofluorometer coupled with a water bath (Julabo MB) to control the temperature. Experiments were performed at 20°C in buffer 10 mM sodium phosphate, 50 mM sodium chloride, pH 7.8, and 5 μM protein concentration for RBP and heterodimers, and 5 μM for the other proteins, with 280 nm as excitation wavelength, 300–500 nm as emission wavelength, and 1 nm bandwidth. Raw signal was normalized for protein concentration.

### 4.7 Analytical size exclusion chromatography coupled to multi angle light scattering (SEC‐MALS)

Analytical SEC measurements were performed coupled to a miniDAWN Multi Angle Light Scattering (MALS) detector and an Optilab refractometer (Wyatt Technology). Samples previously centrifuged and filtered were run in a Superdex 75 Increase 10/300 GL column connected to an Äkta Pure System (GE Healthcare Life Sciences) equilibrated with buffer 10 mM sodium phosphate, 50 mM sodium chloride, 0.02% sodium azide, pH 7.8. Experiments were conducted at room temperature with a protein concentration of 1 and 0.8 mL min^−1^ flow rate. For the samples containing ribose, 0.5 mM of ribose was premixed with protein at 1 mg mL^−1^. Reproducibility during all SEC‐MALS measurements was tested by running a BSA standard at 2 mg mL^−1^ at the beginning and end of all experiments, which resulted in identical data. Determination of weight averaged molar mass was performed by using the Zimm‐Equation with the differential refractive index signal as source for the concentration calculations (refractive index increment dn/dc set to 0.185). Data collection and analysis were done using the ASTRA v.7.3.2 software (Wyatt Technology).

### 4.8 Crystallization and three‐dimensional structure determination

For setting up crystallization assays, protein at 0.5 mM concentration was dialyzed against 20 mM Tris–HCl, 300 mM sodium chloride, pH 7.8. For RBP‐N/RBP‐Trunc heterodimer, 0.5 mM equimolar ratio of each protein was used as initial concentration. Screening plates were set up by a sitting‐drop vapor diffusion method using JCSG Core I‐IV (Qiagen), PEG Suite I‐II (Qiagen), and Additive Screen kits (Hampton Research) in 96 well Intelli plates (Art Robbins Instruments). Plates with 0.8 μL drops in a 1:1, 1:2, and 2:1 protein: mother liquor drop ratio were set up with a nano dispensing crystallization Phoenix robot (Art Robbins Instruments) and stored at 20°C in a hotel‐based crystal imaging system RockImager RI 1000 (Formulatrix). RBP‐N/RBP‐Trunc heterodimer crystals with successful diffraction data were found in 100 mM HEPES pH 7.5, 15% (w/v) PEG 20000 and a drop ratio 1:1. Data were collected at Berlin Electron Storage Ring Society for Synchrotron Radiation beamline 14.2 (BESSY 14.2) operated by the Helmholtz‐Zentrum Berlin using the mxCuBE beamline‐control software (Gabadinho et al., 2010). Measurements at 100 K were performed in a single‐wavelength mode at 0.9184 Å with a PILATUS3S 2M detector (HZB, 2016) in fine‐slicing mode (0.1° wedges). Diffraction images were processed with x‐ray detector software (XDS) and XDSAPP v3.0 (Kabsch, 2010; Sparta et al., 2016). Phasing was performed by molecular replacement with PHASER in the PHENIX software suite v.1.19.2 (Liebschner et al., 2019) using the edited pdb file corresponding to the RBP‐N and RBP‐Trunc halves from *T. maritima* RBP (PDB 2FN9). Data refinement was carried out with phenix.refine (Adams et al., 2010) and iterative manual model building/improvement in COOT v.0.9 (Emsley et al., 2010). Coordinates and structure factors were validated and deposited in the PDB database https://www.rcsb.org/ (Berman et al., 2002) with the accession code: 7PU4. Figures were created with PyMOL Molecular Graphics System v.2.3.0 (Schrodinger, LLC).

### 4.9 Differential scanning calorimetry

Differential scanning calorimetry (DSC) endotherms were collected using a VP‐Capillary DSC instrument (Malvern Panalytical) with a temperature range of 10–130°C and 1.5°C min^−1^ scan rate. Protein samples were prepared at 50 μM after exhaustive dialysis in buffer 10 mM sodium phosphate, 50 mM sodium chloride, pH 7.8, and proper degassing. Instrument equilibration was performed by collecting at least two buffer–buffer scans before each protein‐buffer experiment. Calorimetric reversibility was tested by collecting two consecutive endotherms and calculating the recovery area percentage from the second and first scan, resulting in irreversible thermal‐unfolding transitions for all the constructs reported in the present study. Thermodynamic parameters (*T*_m_ and Δ*H*) were calculated after subtracting physical (buffer–buffer scan) and chemical baselines (heat capacity effects) from each protein‐buffer scan. Thermostabilization by protein–protein interaction (dimer formation) was determined by changes in *T*_m_ and Δ*H* when two different proteins were combined in equimolar concentration. DSC experiments in presence of ribose were performed at 50 μM protein concentration and 0.5 mM ribose premixed in the same working buffer before the heating cycles. Buffer–buffer scans were collected containing the same amount of ribose as protein/ribose‐buffer experiments and subtracted as indicated. Ribose stability at high temperatures was tested and no endotherm distortions were observed in the concentration and temperature ranges assayed. Origin v.7.0 (OriginLab Corporation) with MicroCal software was used for data analysis.

### 4.10 Isothermal titration calorimetry

Binding assays followed by isothermal titration calorimetry (ITC) were performed using a TA Nano ITC low volume device (TA Instruments). Titrations were obtained at 20°C in buffer 10 mM sodium phosphate, 50 mM sodium chloride, pH 7.8, and 100 μM of protein concentration, which was exhaustively dialyzed against the working buffer. Ribose solution was prepared in the same working buffer to minimize dilution heats and was loaded in the syringe at 0.8 mM concentration. Protein and ligand solutions were degassed with a vacuum pump for 90 min before carrying out the experiments, and concentrations were optimized in order to reach *c* values higher than 10. Independent triplicates of ITC experiments were performed with 25 injections of 2 μL volume, spacing of 350 s between injections, and stirring at 300 rpm. Dilution heats were subtracted from the heat associated with each injection to get accurate parameters. Baseline and integration intervals were carefully checked to avoid experiment distortions. Binding constant (*K*_D_), enthalpy change (Δ*H*), and binding stoichiometry (*n*) were determined by nonlinear fitting of normalized data assuming a 1:1 binding model and using TA ITC software. All titration replicates fulfilled the characteristics for an accurate parameter determination that have been analyzed by experimental and simulation data (Turnbull & Daranas, 2003).

## AUTHOR CONTRIBUTIONS

Florian Michel, Sergio Romero‐Romero, and Birte Höcker designed the research, Florian Michel, Sergio Romero‐Romero purified the different constructs, Florian Michel collected CD, IF, and SEC‐MALS data, Sergio Romero‐Romero performed DSC and ITC experiments, Florian Michel, Sergio Romero‐Romero crystallized and solved three‐dimensional structure, Florian Michel, Sergio Romero‐Romero, Birte Höcker analyzed the date and wrote the manuscript.

## FUNDING INFORMATION

This work was supported by the European Research Council (ERC Consolidator Grant 647548 “Protein Lego” to Birte Höcker), the VolkswagenStiftung (grant 94747 to Birte Höcker), and by a fellowship from the Alexander von Humboldt and Bayer Science & Education Foundation (Humboldt‐Bayer Research Fellowship for Postdoctoral Researchers to Sergio Romero‐Romero).

## CONFLICT OF INTEREST STATEMENT

The authors declare that they have no conflicts of interest with the contents of this article.

## Supporting information


**Data S1:** Supporting Information

## Data Availability

All data to support the conclusions of this manuscript are included in the main text and Supporting Information. Coordinates and structure factors have been deposited to the Protein Data Bank (PDB) with accession code: 7PU4 (RBP‐N/RBP‐Trunc heterodimer).

## References

[pro4793-bib-0001] Adams PD , Afonine PV , Bunkóczi G , Chen VB , Davis IW , Echols N , et al. PHENIX: A comprehensive python‐based system for macromolecular structure solution. Acta Crystallogr D Biol Crystallogr. 2010;66(2):213–221. 10.1107/S0907444909052925 PMC2815670 20124702

[pro4793-bib-0002] Aggarwal V , Kulothungan SR , Balamurali MM , Saranya SR , Varadarajan R , Ainavarapu SR . Ligand‐modulated parallel mechanical unfolding pathways of maltose‐binding proteins. J Biol Chem. 2011;286(32):28056–28065. 10.1074/jbc.M111.249045 PMC3151051 21659518

[pro4793-bib-0003] Alva V , Söding J , Lupas AN . A vocabulary of ancient peptides at the origin of folded proteins. elife. 2015;4:e09410. 10.7554/eLife.09410 PMC4739770 26653858

[pro4793-bib-0004] Alvarez‐Carreño C , Gupta RJ , Petrov AS , Williams LD . Creative destruction: new protein folds from old. Proc Natl Acad Sci USA. 2022;119(52):e2207897119. 10.1073/pnas.2207897119 PMC9907106 36534803

[pro4793-bib-0005] Bae JE , Kim IJ , Kim KJ , Nam KH . Crystal structure of a substrate‐binding protein from Rhodothermus marinus reveals a single α/β‐domain. Biochem Biophys Res Commun. 2018;497(1):368–373. 10.1016/j.bbrc.2018.02.086 29432740

[pro4793-bib-0006] Banda‐Vázquez J , Shanmugaratnam S , Rodríguez‐Sotres R , Torres‐Larios A , Höcker B , Sosa‐Peinado A . Redesign of LAOBP to bind novel l‐amino acid ligands. Protein Sci. 2018;27(5):957–968. 10.1002/pro.3403 PMC5916193 29524280

[pro4793-bib-0007] Berman HM , Battistuz T , Bhat TN , Bluhm WF , Bourne PE , Burkhardt K , et al. The Protein Data Bank. Acta Crystallogr D Biol Crystallogr. 2002;58(6 I):899–907. 10.1107/S0907444902003451 12037327

[pro4793-bib-0008] Bermejo GA , Strub MP , Ho C , Tjandra N . Determination of the solution‐bound conformation of an amino acid binding protein by NMR paramagnetic relaxation enhancement: use of a single flexible paramagnetic probe with improved estimation of its sampling space. J Am Chem Soc. 2009;131(27):9532–9537. 10.1021/ja902436g PMC2720827 19583434

[pro4793-bib-0009] Bermejo GA , Strub MP , Ho C , Tjandra N . Ligand‐free open‐closed transitions of periplasmic binding proteins: the case of glutamine‐binding protein. Biochemistry. 2010;49(9):1893–1902. 10.1021/bi902045p PMC2831130 20141110

[pro4793-bib-0010] Berntsson RP , Smits SH , Schmitt L , Slotboom DJ , Poolman B . A structural classification of substrate‐binding proteins. FEBS Lett. 2010;584(12):2606–2617. 10.1016/j.febslet.2010.04.043 20412802

[pro4793-bib-0011] Brandts JF , Hu CQ , Lin LN , Mos MT . A simple model for proteins with interacting domains. Applications to scanning calorimetry data. Biochemistry. 1989;28(21):8588–8596. 10.1021/bi00447a048 2690944

[pro4793-bib-0012] Careaga CL , Sutherland J , Sabeti J , Falke JJ . Large amplitude twisting motions of an interdomain hinge: a disulfide trapping study of the galactose‐glucose binding protein. Biochemistry. 1995;34(9):3048–3055. 10.1021/bi00009a036 PMC2892987 7893717

[pro4793-bib-0013] Chandonia JM , Fox NK , Brenner SE . SCOPe: classification of large macromolecular structures in the structural classification of proteins‐extended database. Nucleic Acids Res. 2019;47(D1):D475–D481. 10.1093/nar/gky1134 PMC6323910 30500919

[pro4793-bib-0014] Chandravanshi M , Tripathi SK , Kanaujia SP . An updated classification and mechanistic insights into ligand binding of the substrate‐binding proteins. FEBS Lett. 2021;595(18):2395–2409. 10.1002/1873-3468.14174 34379808

[pro4793-bib-0015] Cheng H , Liao Y , Schaeffer RD , Grishin NV . Manual classification strategies in the ECOD database. Proteins. 2015;83(7):1238–1251. 10.1002/prot.24818 PMC4624060 25917548

[pro4793-bib-0016] Chu BC , DeWolf T , Vogel HJ . Role of the two structural domains from the periplasmic *Escherichia coli* histidine‐binding protein HisJ. J Biol Chem. 2013;288(44):31409–31422. 10.1074/jbc.M113.490441 PMC3814738 24036119

[pro4793-bib-0017] Clifton BE , Jackson CJ . Ancestral protein reconstruction yields insights into adaptive evolution of binding specificity in solute‐binding proteins. Cell Chem Biol. 2016;23(2):236–245. 10.1016/j.chembiol.2015.12.010 26853627

[pro4793-bib-0018] Cooper A , Nutley MA , Walood A . In: Harding SE , Chowdhry BZ , editors. Differential scanning microcalorimetry. Oxford, New York: Oxford University Press; 2000. p. 287–318.

[pro4793-bib-0019] Copley SD . Evolution of new enzymes by gene duplication and divergence. FEBS J. 2020;287(7):1262–1283. 10.1111/febs.15299 PMC9306413 32250558

[pro4793-bib-0020] Cuneo MJ , Beese LS , Hellinga HW . Ligand‐induced conformational changes in a thermophilic ribose‐binding protein. BMC Struct Biol. 2008;8:50. 10.1186/1472-6807-8-50 PMC2630998 19019243

[pro4793-bib-0021] Das S , Dawson NL , Orengo CA . Diversity in protein domain superfamilies. Curr Opin Genet Dev. 2015;35:40–49. 10.1016/j.gde.2015.09.005 PMC4686048 26451979

[pro4793-bib-0022] Dwyer MA , Hellinga HW . Periplasmic binding proteins: a versatile superfamily for protein engineering. Curr Opin Struct Biol. 2004;14(4):495–504. 10.1016/j.sbi.2004.07.004 15313245

[pro4793-bib-0023] Emsley P , Lohkamp B , Scott WG , Cowtan K . Features and development of Coot. Acta Crystallogr D Biol Crystallogr. 2010;66(Pt 4):486–501. 10.1107/S0907444910007493 PMC2852313 20383002

[pro4793-bib-0024] Farías‐Rico JA , Schmidt S , Höcker B . Evolutionary relationship of two ancient protein superfolds. Nat Chem Biol. 2014;10(9):710–715. 10.1038/nchembio.1579 25038785

[pro4793-bib-0025] Felder CB , Graul RC , Lee AY , Merkle HP , Sadee W . The Venus flytrap of periplasmic binding proteins: an ancient protein module present in multiple drug receptors. AAPS PharmSci. 1999;1(2):E2–E26. 10.1208/ps010202 PMC2761117 11741199

[pro4793-bib-0026] Ferruz N , Lobos F , Lemm D , Toledo‐Patino S , Farías‐Rico JA , Schmidt S , et al. Identification and analysis of natural building blocks for evolution‐guided fragment‐based protein design. J Mol Biol. 2020;432(13):3898–3914. 10.1016/j.jmb.2020.04.013 PMC7322520 32330481

[pro4793-bib-0027] Ferruz N , Michel F , Lobos F , Schmidt S , Höcker B . Fuzzle 2.0: Ligand binding in natural protein building blocks. Front Mol Biosci. 2021;8:715–972. 10.3389/fmolb.2021.715972 PMC8416435 34485385

[pro4793-bib-0028] Fukada H , Sturtevant JM , Quiocho FA . Thermodynamics of the binding of L‐arabinose and of D‐galactose to the L‐arabinose‐binding protein of *Escherichia coli* . J Biol Chem. 1983;258(21):13193–13198. 10.1016/S0021-9258(17)44100-7 6355105

[pro4793-bib-0029] Fukami‐Kobayashi K , Tateno Y , Nishikawa K . Domain dislocation: a change of core structure in periplasmic binding proteins in their evolutionary history. J Mol Biol. 1999;286(1):279–290. 10.1006/jmbi.1998.2454 9931266

[pro4793-bib-0030] Gabadinho J , Beteva A , Guijarro M , Rey‐Bakaikoa V , Spruce D , Bowler MW , et al. MxCuBE: a synchrotron beamline control environment customized for macromolecular crystallography experiments. J Synchrotron Radiat. 2010;17(5):700–707. 10.1107/S0909049510020005 PMC3025540 20724792

[pro4793-bib-0031] Gabler F , Nam SZ , Till S , Mirdita M , Steinegger M , Söding J , et al. Protein sequence analysis using the MPI bioinformatics toolkit. Curr Protoc Bioinformatics. 2020;72(1):e108. 10.1002/cpbi.108 33315308

[pro4793-bib-0032] Ganesh C , Shah AN , Swaminathan CP , Surolia A , Varadarajan R . Thermodynamic characterization of the reversible, two‐state unfolding of maltose binding protein, a large two‐domain protein. Biochemistry. 1997;36(16):5020–5028. 10.1021/bi961967b 9125524

[pro4793-bib-0033] Gouridis G , Muthahari YA , de Boer M , Griffith DA , Tsirigotaki A , Tassis K , et al. Structural dynamics in the evolution of a bilobed protein scaffold. Proc Natl Acad Sci U S A. 2021;118(49):e2026165118. 10.1073/pnas.2026165118 PMC8694067 34845009

[pro4793-bib-0034] Gouridis G , Schuurman‐Wolters GK , Ploetz E , Husada F , Vietrov R , de Boer M , et al. Conformational dynamics in substrate‐binding domains influences transport in the ABC importer GlnPQ. Nat Struct Mol Biol. 2015;22(1):57–64. 10.1038/nsmb.2929 25486304

[pro4793-bib-0035] Greenfield NJ . Using circular dichroism spectra to estimate protein secondary structure. Nat Protoc. 2006;1(6):2876–2890. 10.1038/nprot.2006.202 PMC2728378 17406547

[pro4793-bib-0036] Han JH , Batey S , Nickson AA , Teichmann SA , Clarke J . The folding and evolution of multidomain proteins. Nat Rev Mol Cell Biol. 2007;8(4):319–330. 10.1038/nrm2144 17356578

[pro4793-bib-0037] Helmholtz‐Zentrum Berlin für Materialien und Energie . The MX beamlines BL14.1–3 at BESSY II. JLSRF. 2016;2:A47. 10.17815/jlsrf-2-64

[pro4793-bib-0038] Jeffery CJ . Engineering periplasmic ligand binding proteins as glucose nanosensors. Nano Rev. 2011;2:5743. 10.3402/nano.v2i0.5743 PMC3215197 22110874

[pro4793-bib-0039] Kabsch W . XDS. Acta Crystallogr D Biol Crystallogr. 2010;66(Pt 2):125–132. 10.1107/S0907444909047337 PMC2815665 20124692

[pro4793-bib-0040] Kantaev R , Riven I , Goldenzweig A , Barak Y , Dym O , Peleg Y , et al. Manipulating the folding landscape of a multidomain protein. J Phys Chem B. 2018;122(49):11030–11038. 10.1021/acs.jpcb.8b04834 30088929

[pro4793-bib-0041] Khersonsky O , Tawfik DS . Enzyme promiscuity: a mechanistic and evolutionary perspective. Annu Rev Biochem. 2010;79:471–505. 10.1146/annurev-biochem-030409-143718 20235827

[pro4793-bib-0042] Kreimer DI , Malak H , Lakowicz JR , Trakhanov S , Villar E , Shnyrov VL . Thermodynamics and dynamics of histidine‐binding protein, the water‐soluble receptor of histidine permease. Implications for the transport of high and low affinity ligands. Eur J Biochem. 2000;267(13):4242–4252. 10.1046/j.1432-1033.2000.01470.x 10866829

[pro4793-bib-0043] Kröger P , Shanmugaratnam S , Ferruz N , Schweimer K , Höcker B . A comprehensive binding study illustrates ligand recognition in the periplasmic binding protein PotF. Structure. 2021;29(5):433–443.e4. 10.1016/j.str.2020.12.005 33406388

[pro4793-bib-0044] Kröger P , Shanmugaratnam S , Scheib U , Höcker B . Fine‐tuning spermidine binding modes in the putrescine binding protein PotF. J Biol Chem. 2021;297(6):101419. 10.1016/j.jbc.2021.101419 PMC8666671 34801550

[pro4793-bib-0045] Lewis RJ , Muchová K , Brannigan JA , Barák I , Leonard G , Wilkinson AJ . Domain swapping in the sporulation response regulator SpoOA. J Mol Biol. 2000;297(3):757–770. 10.1006/jmbi.2000.3598 10731426

[pro4793-bib-0046] Liebschner D , Afonine PV , Baker ML , Bunkóczi G , Chen VB , Croll TI , et al. Macromolecular structure determination using x‐rays, neutrons and electrons: recent developments in Phenix. Acta Crystallogr D Biol Crystallogr. 2019;75(Pt 10):861–877. 10.1107/S2059798319011471 PMC6778852 31588918

[pro4793-bib-0077] Liu K , Chen, X , & Kaiser, CM (2019). Energetic dependencies dictate folding mechanism in a complex protein. Proceedings of the National Academy of Sciences of the United States of America, 116(51), 25641–25648. 10.1073/pnas.1914366116 PMC6925980 31776255

[pro4793-bib-0047] Louie GV . Porphobilinogen deaminase and its structural similarity to the bidomain binding proteins. Curr Opin Struct Biol. 1993;3:401–408. 10.1016/S0959-440X(05)80113-7

[pro4793-bib-0048] Matilla MA , Ortega Á , Krell T . The role of solute binding proteins in signal transduction. Comput Struct Biotechnol J. 2021;19:1786–1805. 10.1016/j.csbj.2021.03.029 PMC8050422 33897981

[pro4793-bib-0049] Medintz IL , Deschamps JR . Maltose‐binding protein: a versatile platform for prototyping biosensing. Curr Opin Biotechnol. 2006;17(1):17–27. 10.1016/j.copbio.2006.01.002 16413768

[pro4793-bib-0050] Michalska K , Kowiel M , Bigelow L , Endres M , Gilski M , Jaskolski M , et al. 3D domain swapping in the TIM barrel of the α subunit of Streptococcus pneumoniae tryptophan synthase. Acta Crystallogr D Biol Crystallogr. 2020;76(Pt 2):166–175. 10.1107/S2059798320000212 PMC7008512 32038047

[pro4793-bib-0051] Michel F , Shanmugaratnam S , Romero‐Romero S , Höcker B . Structures of permuted halves of a modern ribose‐binding protein. Acta Crystallogr D Biol Crystallogr. 2023;79(Pt 1):40–49. 10.1107/S205979832201186X PMC9815098 36601806

[pro4793-bib-0052] Nepomnyachiy S , Ben‐Tal N , Kolodny R . Complex evolutionary footprints revealed in an analysis of reused protein segments of diverse lengths. Proc Natl Acad Sci U S A. 2017;114(44):11703–11708. 10.1073/pnas.1707642114 PMC5676897 29078314

[pro4793-bib-0053] Ortega G , Castaño D , Diercks T , Millet O . Carbohydrate affinity for the glucose‐galactose binding protein is regulated by allosteric domain motions. J Am Chem Soc. 2012;134(48):19869–19876. 10.1021/ja3092938 23148479

[pro4793-bib-0054] Paithankar KS , Enderle M , Wirthensohn DC , Miller A , Schlesner M , Pfeiffer F , et al. Structure of the archaeal chemotaxis protein CheY in a domain‐swapped dimeric conformation. Acta Crystallogr F: Struct Biol Commun. 2019;75, (Pt 9):576–585. 10.1107/S2053230X19010896 PMC6718144 31475924

[pro4793-bib-0055] Pistolesi S , Tjandra N , Bermejo GA . Solution NMR studies of periplasmic binding proteins and their interaction partners. Biomol Concepts. 2011;2(1–2):53–64. 10.1515/bmc.2011.005 PMC5506692 25962019

[pro4793-bib-0056] Prajapati RS , Indu S , Varadarajan R . Identification and thermodynamic characterization of molten globule states of periplasmic binding proteins. Biochemistry. 2007;46(36):10339–10352. 10.1021/bi700577m 17696409

[pro4793-bib-0057] Privalov PL . Stability of proteins: small globular proteins. Adv Protein Chem. 1979;33:167–241. 10.1016/s0065-3233(08)60460-x 44431

[pro4793-bib-0058] Ribeiro LF , Amarelle V , Ribeiro LFC , Guazzaroni ME . Converting a periplasmic binding protein into a synthetic biosensing switch through domain insertion. BioMed Res Int. 2019;2019:4798793 10.1155/2019/4798793. 10.1155/2019/4798793 PMC6335823 30719443

[pro4793-bib-0059] Romero‐Romero S , Kordes S , Michel F , Höcker B . Evolution, folding, and design of TIM barrels and related proteins. Curr Opin Struct Biol. 2021;68:94–104. 10.1016/j.sbi.2020.12.007 PMC8250049 33453500

[pro4793-bib-0060] Scheepers GH , Nijeholt JALA , Poolman B . An updated structural classification of substrate‐binding proteins. FEBS Lett. 2016;590(23):4393–4401. 10.1002/1873-3468.12445 27714801

[pro4793-bib-0061] Scheib U , Shanmugaratnam S , Farías‐Rico JA , Höcker B . Change in protein‐ligand specificity through binding pocket grafting. J Struct Biol. 2014;185(2):186–192. 10.1016/j.jsb.2013.06.002 23792166

[pro4793-bib-0062] Schreier B , Stumpp C , Wiesner S , Höcker B . Computational design of ligand binding is not a solved problem. Proc Natl Acad Sci U S A. 2009;106(44):18491–18496. 10.1073/pnas.0907950106 PMC2773959 19833875

[pro4793-bib-0063] Sillitoe I , Bordin N , Dawson N , Waman VP , Ashford P , Scholes HM , et al. CATH: increased structural coverage of functional space. Nucleic Acids Res. 2021;49(D1):D266–D273. 10.1093/nar/gkaa1079 PMC7778904 33237325

[pro4793-bib-0064] Smaldone G , Ruggiero A , Balasco N , Vitagliano L . Development of a protein scaffold for arginine sensing generated through the dissection of the arginine‐binding protein from *Thermotoga maritima* . Int J Mol Sci. 2020;21(20):7503. 10.3390/ijms21207503 PMC7589609 33053818

[pro4793-bib-0065] Sparta KM , Krug M , Heinemann U , Mueller U , Weiss MS . XDSAPP2.0. J Appl Crystallogr. 2016;49:1085–1092. 10.1107/S1600576716004416

[pro4793-bib-0066] Steffen V , Otten J , Engelmann S , Radek A , Limberg M , Koenig BW , et al. A toolbox of genetically encoded FRET‐based biosensors for rapid l‐lysine analysis. Sensors. 2016;16(10):1604. 10.3390/s16101604 PMC5087393 27690044

[pro4793-bib-0067] Structural Genomics Consortium , China Structural Genomics Consortium , Northeast Structural Genomics Consortium , Gräslund S , Nordlund P , Weigelt J , et al. Protein production and purification. Nat Methods. 2008;5(2):135–146. 10.1038/nmeth.f.202 PMC3178102 18235434

[pro4793-bib-0068] Tawfik DS . Enzyme promiscuity and evolution in light of cellular metabolism. FEBS J. 2020;287(7):1260–1261. 10.1111/febs.15296 32250557

[pro4793-bib-0069] Toledo‐Patiño S , Chaubey M , Coles M , Höcker B . Reconstructing the remote origins of a fold singleton from a Flavodoxin‐like ancestor. Biochemistry. 2019;58(48):4790–4793. 10.1021/acs.biochem.9b00900 PMC6968885 31724394

[pro4793-bib-0070] Tullman J , Nicholes N , Dumont MR , Ribeiro LF , Ostermeier M . Enzymatic protein switches built from paralogous input domains. Biotechnol Bioeng. 2016;113(4):852–858. 10.1002/bit.25852 26461040

[pro4793-bib-0071] Turnbull WB , Daranas AH . On the value of c: can low affinity systems be studied by isothermal titration calorimetry? J Am Chem Soc. 2003;125(48):14859–14866. 10.1021/ja036166s 14640663

[pro4793-bib-0072] Vergara R , Berrocal T , Juárez Mejía EI , Romero‐Romero S , Velázquez‐López I , Pulido NO , et al. Thermodynamic and kinetic analysis of the LAO binding protein and its isolated domains reveal non‐additivity in stability, folding and function. FEBS J. 2023;290:4496–4512. 10.1111/febs.16819 37178351

[pro4793-bib-0073] Vergara R , Romero‐Romero S , Velázquez‐López I , Espinoza‐Pérez G , Rodríguez‐Hernández A , Pulido NO , et al. The interplay of protein‐ligand and water‐mediated interactions shape affinity and selectivity in the LAO binding protein. FEBS J. 2020;287(4):763–782. 10.1111/febs.15019 31348608

[pro4793-bib-0074] Vogel C , Bashton M , Kerrison ND , Chothia C , Teichmann SA . Structure, function and evolution of multidomain proteins. Curr Opin Struct Biol. 2004;14(2):208–216. 10.1016/j.sbi.2004.03.011 15093836

[pro4793-bib-0075] Wenk M , Jaenicke R , Mayr EM . Kinetic stabilisation of a modular protein by domain interactions. FEBS Lett. 1998;438(1–2):127–130. 10.1016/s0014-5793(98)01287-3 9821973

[pro4793-bib-0076] Xia Y , Li K , Li J , Wang T , Gu L , Xun L . T5 exonuclease‐dependent assembly offers a low‐cost method for efficient cloning and site‐directed mutagenesis. Nucleic Acids Res. 2019;47(3):e15. 10.1093/nar/gky1169 PMC6379645 30462336

