# Supplementary material for: Retracing the evolution of a modern periplasmic binding protein
Source: Protein Sci. 2023 Nov 1;32(11):e4793. doi: 10.1002/pro.4793 (PMC10601554; doi:10.1002/pro.4793)
Supplement: Supplementary file 1 — Data S1: Supporting Information [file PRO-32-e4793-s001.pdf]

Supplementary Information for:

Retracing the evolution of a modern periplasmic binding protein

Florian Michel,[1†] Sergio Romero-Romero,[1†] Birte Höcker[1*]

[1] Department of Biochemistry, University of Bayreuth, Bayreuth 95447, Germany.

[†] These authors contributed equally to the work.

Correspondence

* Corresponding author. Birte Höcker. Department of Biochemistry, University of Bayreuth, Bayreuth 95447, Germany. Phone: +490921557845. **E-mail:** birte.hoecker@uni-bayreuth.de

This file includes:

- Supplementary figures 1-7.
- Supplementary tables 1-4.

Supplementary figures and tables

List of Supplementary Figures:

- **Figure S1.** Representative HHpred results for the RBP sequence.
- **Figure S2**. Intrinsic fluorescence measurements of the first- and second-generation constructs.
- **Figure S3**. DSC experiments for the first- and second-generation constructs.
- **Figure S4**. SDS-PAGE of RBP, the individual first-generation halves, and the mixed heterodimer.
- **Figure S5**. Crystallographic dimer formed by the asymmetric-unit mate of RBP-N/RBP-Trunc heterodimer crystal structure.
- **Figure S6**. Biophysical characterization of the second-generation constructs.
- **Figure S7**. DSC endotherms for the co-expressed RBP-$N_{N\text{-His}}$/RBP-$C_{N\text{-Strep}}$ heterodimer.

List of Supplementary Tables:

- **Table S1.** Amino acid sequences of the proteins analyzed in this work.
- **Table S2.** Obtained values from the SEC-MALS measurements for the different RBP constructs.
- **Table S3.** DSC thermodynamic parameters (T_m and ΔH) for the different RBP constructs in absence and presence of ribose.
- **Table S4.** Data collection and refinement statistics for crystal structures.

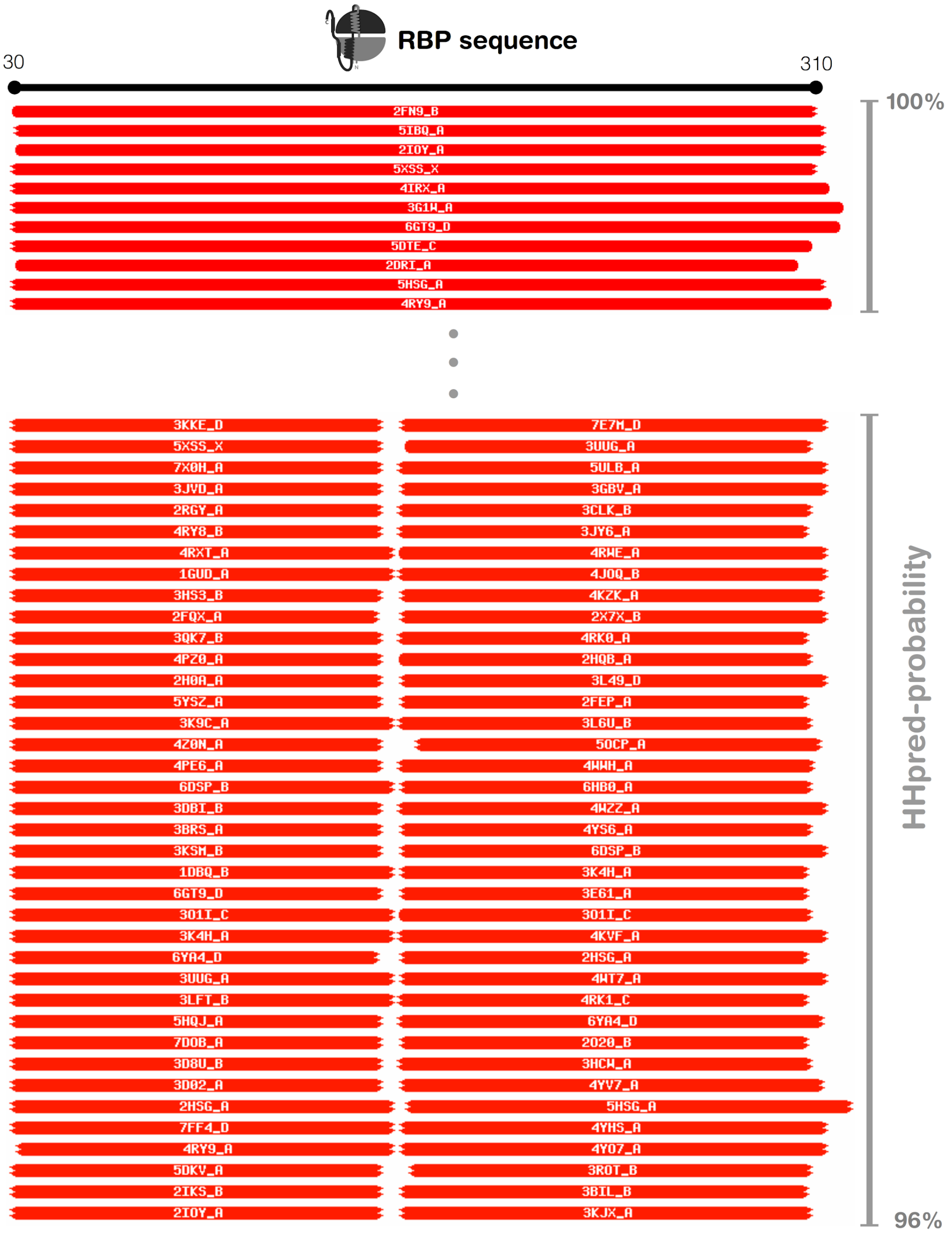

Figure S1. Representative HHpred results for the RBP sequence. Visualization of the HHpred output showing the query sequence as a black bar. The database matches are shown as red horizontal bars underneath with their respective identifiers. Bar length is indicating its coverage with respect to the query and is colored according to its significance (red as very significant to orange, yellow, green and cyan as less significant). Top and longer bars show the alignment of other full-length PBPs on the query sequence while bottom and shorter bars indicate the alignment of the individual lobes. On the right is the HHpred probability shown for the presented sequence range. Numbering has been adapted to be consistent with uniprot entry Q9X053.

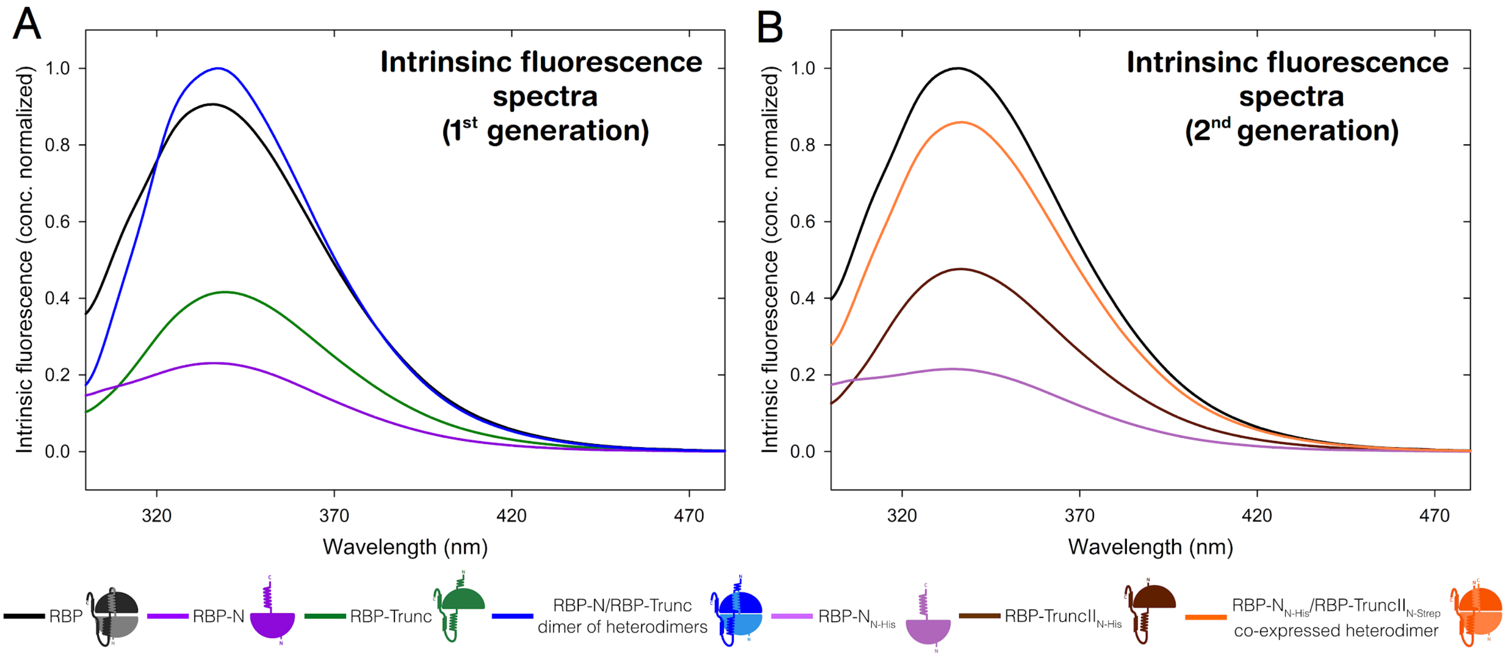

Figure S2. Intrinsic fluorescence measurements of the first- and second-generation constructs. Fluorescence spectra measured from 300-480 nm at an excitation wavelength of 280 nm of RBP (black), RBP-N (violet), RBP-Trunc (green) and the mixed RBP-N/RBP-Trunc heterodimer (**A**) and RBP-$N_{N\text{-}His}$ (red), RBP-$TruncII_{N\text{-}His}$ (brown) and the co-expressed RBP-$N_{N\text{-}His}$/RBP-$TruncII_{N\text{-}Strep}$ heterodimer (**B**) in 10 mM sodium phosphate, 50 mM sodium chloride, pH 7.8. Signal was normalized by protein concentration.

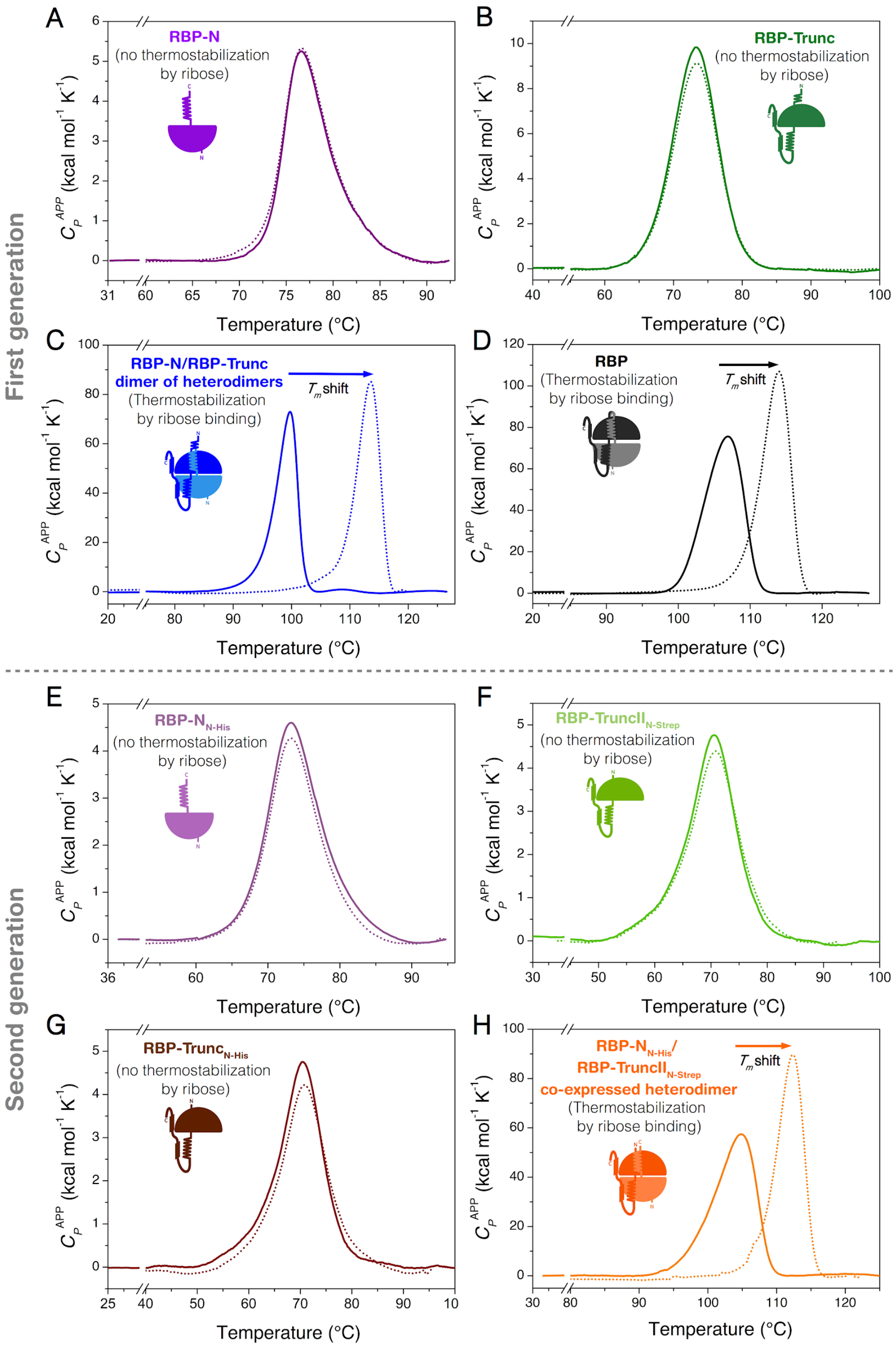

Figure S3. DSC experiments for the first- and second-generation constructs. DSC endotherms at 1.5 °C min^{-1} without ribose (solid lines) and with 0.5 mM ribose (dotted lines) of **(A)** RBP-N (violet), **(B)** RBP-Trunc (green), **(C)** RBP-N/RBP-Trunc heterodimer, **(D)** full-length RBP (black), **(E)** RBP-N$_{N\text{-His}}$ (light purple), **(F)** RBP-TruncII$_{N\text{-Strep}}$ (light green), **(G)** RBP-TruncII$_{N\text{-His}}$ (brown), and **(H)** co-expressed RBP-N$_{N\text{-His}}$/RBP-TruncII$_{N\text{-Strep}}$ heterodimer (orange). Experiments were performed in 10 mM sodium phosphate, 50 mM sodium chloride, pH 7.8 and the physical and chemical baselines have been subtracted.

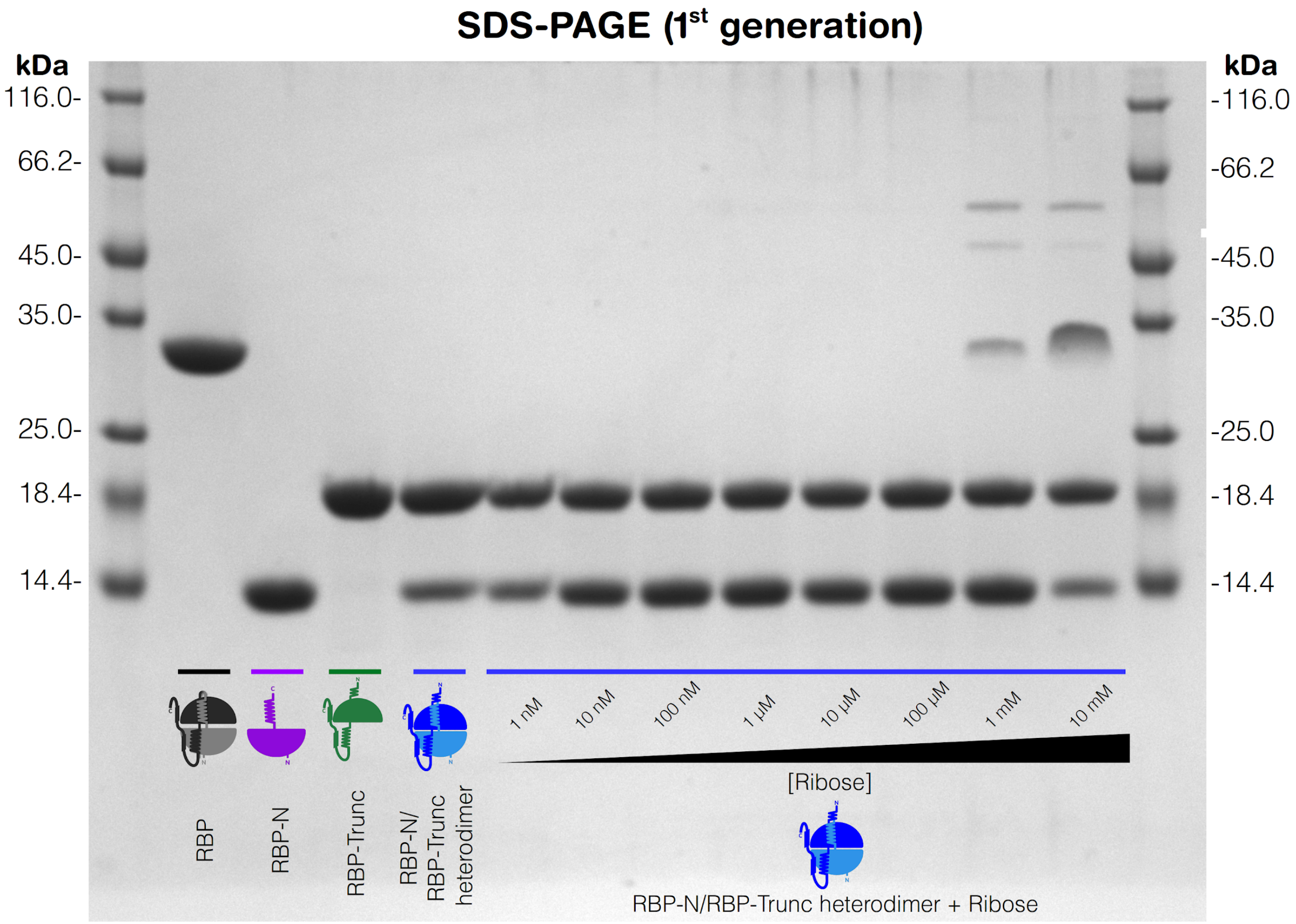

Figure S4. SDS-PAGE of RBP, the individual first-generation halves, and the mixed heterodimer. Purified RBP, RBP-N, RBP-Trunc and RBP-N/RBP-Trunc heterodimer (lane 2-5 respectively) show single proteins at the expected molecular weight without major contaminants. Addition of ribose to the heterodimer appears to stabilise the complex to a degree where it becomes resistant to dissociation in the SDS loading buffer and subsequent heating as indicated by the presence of higher oligomer bands in the presence of $\geq$1 mM [ribose] (lanes 6-13). Molecular weight has been estimated as indicated by the addition of the molecular weight standard (lane 1 and 14, weights annotated).

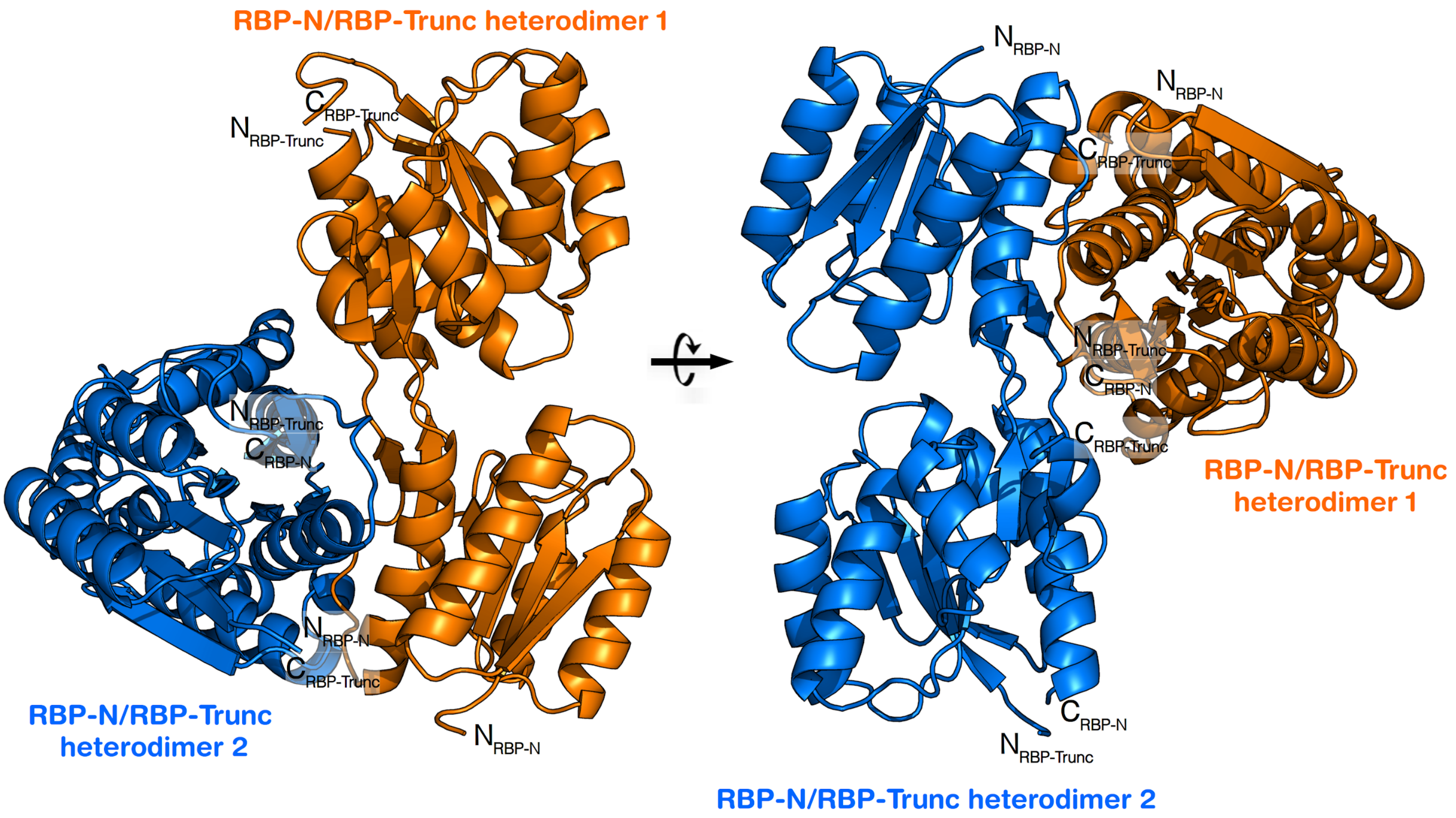

Figure S5. Crystallographic dimer formed by the asymmetric-unit mate of RBP-N/RBP-Trunc heterodimer crystal structure. Each heterodimer is indicated in orange and blue.

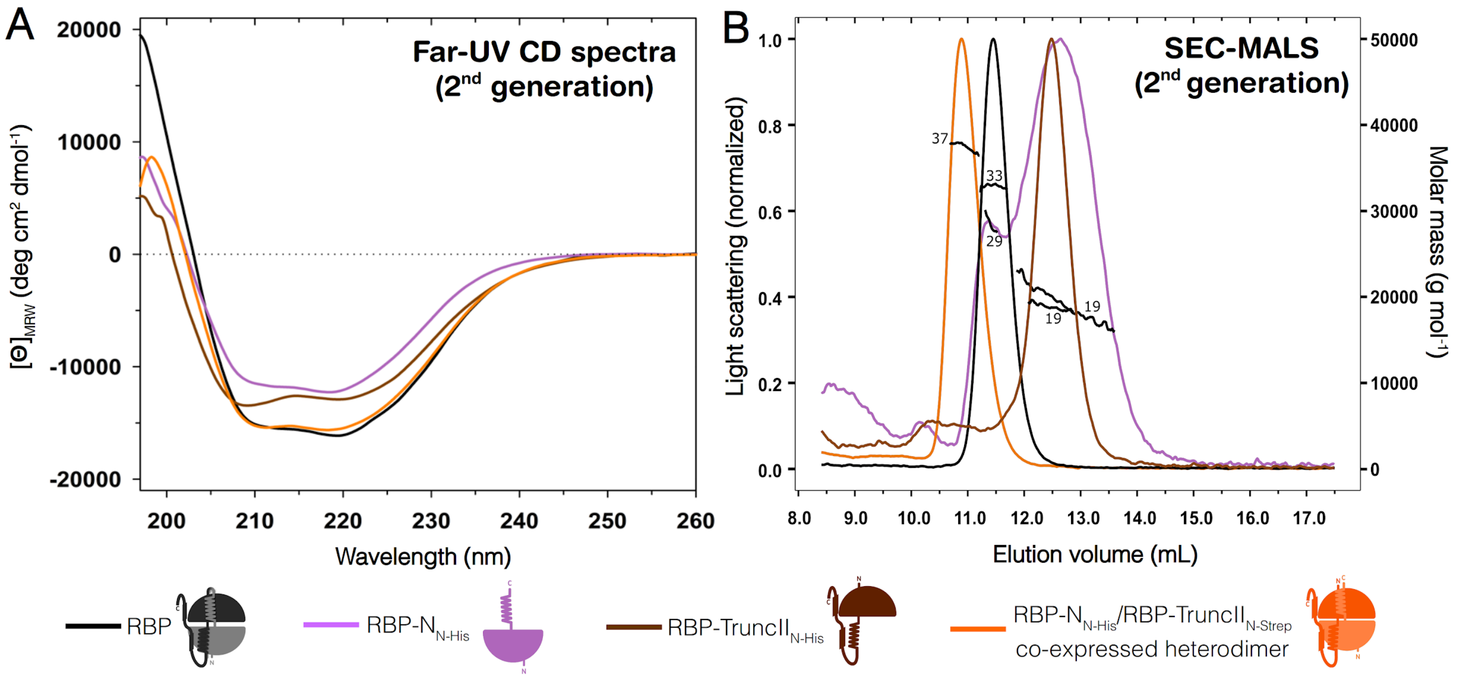

Figure S6. Biophysical characterization of the second-generation constructs. (A) Far-UV CD spectra of RBP (black), RBP-$N_{N\text{-His}}$ (light purple), RBP-$TruncII_{N\text{-His}}$ (brown) and the co-expressed RBP-$N_{N\text{-His}}$/RBP-$TruncII_{N\text{-Strep}}$ heterodimer (orange) in 10 mM sodium phosphate, 50 mM sodium chloride, pH 7.8. **(B)** SEC-MALS measurements in 10 mM sodium phosphate, 50 mM sodium chloride, 0.02% sodium azide, pH 7.8. Numbers indicate the determined molecular weight after data analysis. Values derived from the experiments are reported in Supplementary Table S2.

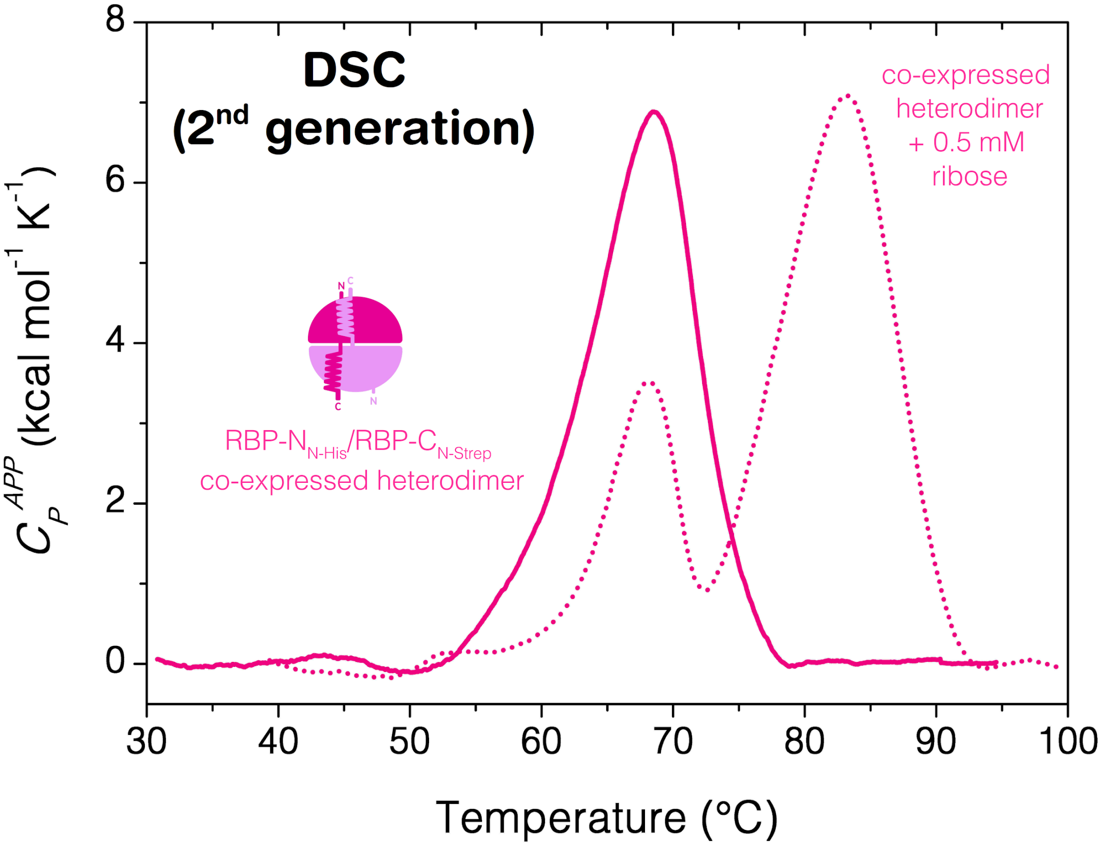

Figure S7. DSC endotherms for the co-expressed RBP-N$_{N\text{-}His}$/RBP-C$_{N\text{-}Strep}$ heterodimer. DSC experiments were collected at 1.5 °C min^{-1} without ribose (solid lines) and with 0.5 mM ribose (dotted lines) in 10 mM sodium phosphate, 50 mM sodium chloride, pH 7.8. Physical and chemical baselines were subtracted.

Table S1. Amino acid sequences of the proteins analyzed in this work. Tags used for expression/purification are highlighted in red. Differences in constructs (numbering consistent with uniprot entry Q9X053) as indicated below. RBP-N & RBP-N$_{\text{N-His}}$: correspond to the N-terminal lobe (30-153); RBP-C: corresponding to the flavodoxin-like architecture derived from the RBP C-terminal half, vestigial helix on N- and additional elements on C-terminus removed (157-291); RBP-Trunc: derived from the alternate initiation of translation at M142 (142-310); RBP-TruncII$_{\text{N-His/N-Strep}}$: corresponds to truncated construct, with the vestigial helix at the new N-terminus removed (156-310); RBP-C$_{\text{N-Strep}}$: corresponds to the C-terminal half, additional residues added of C-terminus (156-294).

Protein		Representation	Expression tag	Sequence
First generation	RBP	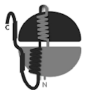	His-tag (C-terminal)	MKGKMAIVISTLNNPWFVVLAETAKQRAEQLGYEATIFDSQNDTAKESAHFDAIIAAG<br>YDAIIFNPTDADGSIANVKRAKEAGIPVFCVDRGINARGLAVAQIYSDNYYGGVLAGE<br>YFVKFLKEKYPDAKEIPYAELLGILSAQPTWDRSNGFHSVVDQYPEFKMVAQQSAEFD<br>RDTAYKVTEQILQAHPEIKAIWCGNDAMALGAMKACEAAGRTDIYIFGFDGAEDVINA<br>IKEGKQIVATIMQFPKLMARLAVEWADQYLRGERSFPEIVPVTVELVTRENIDKYTAY<br>GRK**LEHHHHHH**
	RBP-N	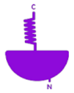	His-tag (C-terminal)	MKGKMAIVISTLNNPWFVVLAETAKQRAEQLGYEATIFDSQNDTAKESAHFDAIIAAG<br>YDAIIFNPTDADGSIANVKRAKEAGIPVFCVDRGINARGLAVAQIYSDNYYGGVLMGE<br>YFVKFLKEK**LEHHHHHH**
	RBP-C	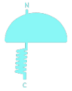	His-tag (C-terminal)	MKEIPYAELLGILSAQPTWDRSNGFHSVVDQYPEFKMVAQQSAEFDRDTAYKVTEQIL<br>QAHPEIKAIWCGNDAMALGAMKACEAAGRTDIYIFGFDGAEDVINAIKEGKQIVATIM<br>QFPKLMARLAVEWADQYLR**LEHHHHHH**
	RBP-Trunc	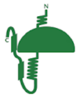	His-tag (C-terminal)	MGEYFVKFLKEKYPDAKEIPYAELLGILSAQPTWDRSNGFHSVVDQYPEFKMVAQQSA<br>EFDRDTAYKVTEQILQAHPEIKAIWCGNDAMALGAMKACEAAGRTDIYIFGFDGAEDV<br>INAIKEGKQIVATIMQFPKLMARLAVEWADQYLRGERSFPEIVPVTVELVTRENIDKY<br>TAYGRK**LEHHHHHH**
Second generation	RBP-N$_{\text{N-His}}$	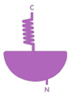	His-tag (N-terminal + TEV site)	**MHHHHHHGLENLYFQGLE**KGKMAIVISTLNNPWFVVLAETAKQRAEQLGYEATIFDSQ<br>NDTAKESAHFDAIIAAGYDAIIFNPTDADGSIANVKRAKEAGIPVFCVDRGINARGLA<br>VAQIYSDNYYGGVLMGEYFVKFLKEK
	RBP-C$_{\text{N-Strep}}$	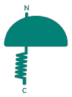	Strep-tag (N-terminal + TEV site)	**MWSHPQFEKGLENLYFQGLE**DAKEIPYAELLGILSAQPTWDRSNGFHSVVDQYPEFKM<br>VAQQSAEFDRDTAYKVTEQILQAHPEIKAIWCGNDAMALGAMKACEAAGRTDIYIFGF<br>DGAEDVINAIKEGKQIVATIMQFPKLMARLAVEWADQYLRGER
	RBP-TruncII$_{\text{N-Strep}}$	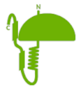	Strep-tag (N-terminal + TEV site)	**MWSHPQFEKGLENLYFQGLE**DAKEIPYAELLGILSAQPTWDRSNGFHSVVDQYPEFKM<br>VAQQSAEFDRDTAYKVTEQILQAHPEIKAIWCGNDAMALGAMKACEAAGRTDIYIFGF<br>DGAEDVINAIKEGKQIVATIMQFPKLMARLAVEWADQYLRGERSFPEIVPVTVELVTR<br>ENIDKYTAYGRK
	RBP-TruncII$_{\text{N-His}}$	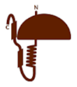	His-tag (N-terminal + TEV site)	**MHHHHHHGLENLYFQGLE**DAKEIPYAELLGILSAQPTWDRSNGFHSVVDQYPEFKMVA<br>QQSAEFDRDTAYKVTEQILQAHPEIKAIWCGNDAMALGAMKACEAAGRTDIYIFGFDG<br>AEDVINAIKEGKQIVATIMQFPKLMARLAVEWADQYLRGERSFPEIVPVTVELVTREN<br>IDKYTAYGRK

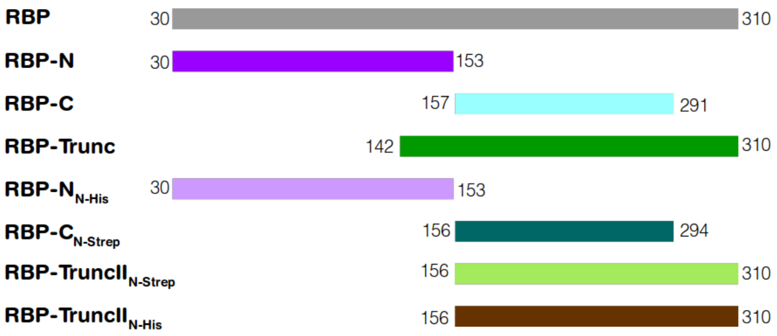

Table S2. Obtained values from the SEC-MALS measurements for the different RBP constructs.

Protein			Expected Mw (kDa)	Experimental Mw (kDa)	Polydispersity (Mw/Mn)‡	Mass Fraction (%)	Oligomeric state
**Individual constructs**							
RBP		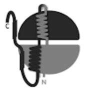	33.6	32.9 ± 0.1	1.000 ± 0.005	100	Monomer
RBP + 0.5 mM ribose				32.7 ± 0.2	1.000 ± 0.008	100	Monomer
RBP-N		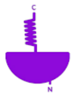	14.7	15.3 ± 0.2	1.001 ± 0.022	100	Monomer
RBP-Trunc	Peak 1	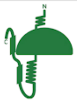	21.5	21.0 ± 0.2	1.000 ± 0.011	90.1	Monomer
	Peak 2			43.1 ± 0.7	1.000 ± 0.031	9.9	Homodimer
RBP-N$_{N\text{-His}}$	Peak 1	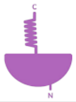	15.7	19.1 ± 0.2	1.003 ± 0.016	86.5	Monomer
	Peak 2			29.1 ± 0.5	1.002 ± 0.025	13.5	Homodimer
RBP-TruncII$_{N\text{-His}}$		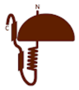	20.9	18.8 ± 0.2	1.000 ± 0.015	100	Monomer
**Heterodimers**							
RBP-N/RBP-Trunc mixed heterodimer		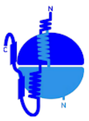	36.2	69.8 ± 0.2	1.001 ± 0.005	100	Dimer of heterodimers
RBP-N/RBP-Trunc mixed heterodimer + 0.5 mM ribose	Peak 1		36.2	43.1 ± 0.2	1.001 ± 0.006	87.3	Heterodimer
	Peak 2			73.1 ± 0.7	1.000 ± 0.012	12.7	Dimer of heterodimers
RBP-N$_{N\text{-His}}$/RBP-TruncII$_{N\text{-Strep}}$ co-expressed heterodimer		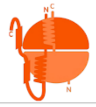	36.8	37.3 ± 0.2	1.000 ± 0.007	100	Heterodimer

± indicates the standard deviation of 3 separate runs.

‡ Polydispersity was calculated by M_w/M_n; M_w - weight-average molar mass moment measured by light scattering; M_n - number-average molar mass moment. A ratio M_w/M_n=1 indicates a homogeneous (i.e., monodisperse) sample, because the average mass is independent of the averaging method.

Table S3. DSC thermodynamic parameters (T_m and ΔH) for the different RBP constructs in absence and presence of ribose.

Protein		T_m (°C)	ΔH (kcal mol^{-1})	Interaction with ribose‡
**Individual constructs**				
RBP	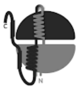	106.9 ± 0.4	531.4 ± 1.2	Yes
RBP + 0.5 mM ribose		114.0 ± 0.9	553.9 ± 2.0	
RBP-N	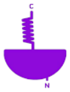	76.6 ± 0.2	35.2 ± 0.3	No
RBP-N + 0.5 mM ribose		76.7 ± 0.3	35.9 ± 0.6	
RBP-Trunc	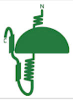	73.3 ± 0.1	79.9 ± 0.4	No
RBP-Trunc + 0.5 mM ribose		73.4 ± 0.2	75.0 ± 1.3	
RBP-$N_{N\text{-}His}$	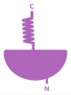	73.2 ± 0.1	42.9 ± 0.4	No
RBP-$N_{N\text{-}His}$ + 0.5 mM ribose		73.1 ± 0.2	38.7 ± 1.1	
RBP-$TruncII_{N\text{-}Strep}$	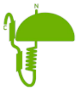	70.6 ± 0.2	54.4 ± 0.9	No
RBP-$TruncII_{N\text{-}Strep}$ + 0.5 mM ribose		70.8 ± 0.3	49.9 ± 1.4	
RBP-$TruncII_{N\text{-}His}$	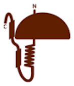	70.4 ± 0.4	50.3 ± 0.5	No
RBP-$TruncII_{N\text{-}His}$ + 0.5 mM ribose		70.9 ± 0.5	44.9 ± 0.8	
**Heterodimers**				
RBP-N/RBP-Trunc mixed heterodimer	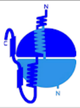	99.7 ± 0.3	355.7 ± 0.9	Yes
RBP-N/RBP-Trunc mixed heterodimer + 0.5 mM ribose		113.5 ± 0.4	484.8 ± 1.6	
RBP-$N_{N\text{-}His}$/RBP-$TruncII_{N\text{-}Strep}$ co-expressed heterodimer	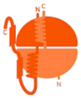	104.8 ± 0.3	462.7 ± 2.3	Yes
RBP-$N_{N\text{-}His}$/RBP-$TruncII_{N\text{-}Strep}$ co-expressed heterodimer + 0.5 mM ribose		113.9 ± 0.4	496.1 ± 1.8	
RBP-$N_{N\text{-}His}$/RBP-$C_{N\text{-}Strep}$ co-expressed heterodimer	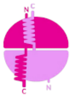	68.4 ± 0.5	73.9 ± 0.6	Yes
RBP-$N_{N\text{-}His}$/RBP-$C_{N\text{-}Strep}$ co-expressed heterodimer + 0.5 mM ribose		1st peak: 68.2 ± 0.7<br>2nd peak: 83.5 ± 0.9	1st peak: 19.2 ± 1.3<br>2nd peak: 83.4 ± 0.8	

‡ Interaction with ribose was determined by changes in thermostability (T_m) and enthalpy (ΔH) parameters comparing DSC endotherms collected with and without 0.5 mM ribose.

Table S4. Data collection and refinement statistics for crystal structures. Statistics for the highest resolution shell are shown in brackets.

Protein	RBP-N/RBP-Trunc heterodimer
PDB ID	7PU4
Wavelength (Å)	0.9184
Resolution range	39.01 – 1.69 (1.75 – 1.69)
Space group	P 21 21 21
Unit cell [a, b, c (Å) / α, β, γ (°)]	65.2 84.2 103.8 / 90 90 90
Total reflections	859968 (81810)
Unique reflections	64629 (6298)
Multiplicity	13.3 (12.8)
Completeness (%)	99.76 (98.39)
Mean I/sigma(I)	14.25 (0.96)
Wilson B-factor	30.6
R-merge	0.129 (1.837)
R-meas	0.127 (1.196)
R-pim	0.035 (1.104)
CC1/2	0.999 (0.318)
CC*	1.000 (0.695)
Matthews coefficient V_m (Å^3 Da^{-1})	1.97
Solvent content (%)	37.7
Protein molecules per asymmetric unit	4 halves
Reflections used in refinement	64515 (6294)
Reflections used for R-free	2095 (205)
R-work	0.206 (0.460)
R-free	0.240 (0.516)
CC(work)	0.964 (0.612)
CC(free)	0.956 (0.581)
Number of non-hydrogen atoms	4757
macromolecules	4373
ligands	0
solvent	384
Protein residues	574
RMS(bonds)	0.008
RMS(angles)	0.900
Ramachandran favored (%)	97.88
Ramachandran allowed (%)	1.77
Ramachandran outliers (%)	0.35
Rotamer outliers (%)	0.00
Clashscore	3.75
Average B-factor	43.1
macromolecules	43.0
solvent	44.6
Number of TLS groups	4
